# An Azomethine Derivative, BCS3, Targets XIAP and cIAP1/2 to Arrest Breast Cancer Progression Through MDM2-p53 and Bcl-2-Caspase Signaling Modulation

**DOI:** 10.3390/ph17121645

**Published:** 2024-12-06

**Authors:** Reetuparna Acharya, Pran Kishore Deb, Katharigatta N. Venugopala, Shakti Prasad Pattanayak

**Affiliations:** 1 Division of Advanced Pharmacology, Department of Pharmaceutical Sciences and Technology, Birla Institute of Technology (BIT), Mesra, Ranchi 835215, India; phdph10051.18@bitmesra.ac.in; 2 Department of Pharmaceutical Sciences and Technology, Birla Institute of Technology (BIT), Mesra, Ranchi 835215, India; 3 Department of Pharmaceutical Sciences, College of Clinical Pharmacy, King Faisal University, Al-Ahsa 31982, Saudi Arabia; kvenugopala@kfu.edu.sa; 4 Department of Biotechnology and Food Science, Faculty of Applied Sciences, Durban University of Technology, Durban 4001, South Africa; 5 Department of Biochemistry, School of Medicine, Case Western Reserve University, Woods Building, W437, 2109 Adelbert Road, Cleaveland, OH 44106, USA

**Keywords:** breast cancer, caspases, 7,12-dimethylbenz(a)anthracene, apoptosis, inhibitors of apoptosis protein, molecular docking

## Abstract

**Background**: Breast cancer influences more than 2 million women worldwide annually. Since apoptotic dysregulation is a cancer hallmark, targeting apoptotic regulators encompasses strategic drug development for cancer therapy. One such class of apoptotic regulators is inhibitors of apoptosis proteins (IAP) which are a class of E3 ubiquitin ligases that actively function to support cancer growth and survival. **Methods**: The current study reports design, synthesis, docking analysis (based on binding to IAP-BIR3 domains), anti-proliferative and anti-tumor potential of the azomethine derivative, 1-(4-chlorophenyl)-N-(4-ethoxyphenyl)methanimine (**BCS3**) on breast cancer (in vitro and in vivo) and its possible mechanisms of action. **Results**: Strong selective cytotoxic activity was observed in MDA-MB-231, MCF-7, and MDA-MB-468 breast cancer cell lines that exhibited IC50 values, 1.554 µM, 5.979 µM, and 6.462 µM, respectively, without affecting normal breast cells, MCF-10A. For the evaluation of the cytotoxic potential of **BCS3**, immunofluorescence, immunoblotting, and FACS (apoptosis and cell cycle) analyses were conducted. **BCS3** antagonized IAPs, thereby causing MDM2-p53 and Bcl-2-Caspase-mediated intrinsic and extrinsic apoptosis. It also modulated p53 expression causing p21-CDK1/cyclin B1-mediated cell cycle arrest at S and G2/M phases. The in vitro findings were consistent with in vivo findings as observed by reduced tumor volume and apoptosis initiation (TUNEL assay) by IAP downregulation. **BCS3** also produced potent synergistic effects with doxorubicin on tumor inhibition. **Conclusions**: Having witnessed the profound anti-proliferative potential of **BCS3**, the possible adverse effects related to anti-cancer therapy were examined following OECD 407 guidelines which confirmed its systemic safety profile and well tolerability. The results indicate the promising effect of **BCS3** as an IAP antagonist for breast cancer therapy with fewer adverse effects.

## 1. Introduction

Breast cancer is the second most recognized cancer among all cancers worldwide after lung cancer, and the incidences are promptly increasing with time [1]. The global report of the World Health Organization classifies breast cancer as the most prevailing malignancy worldwide, with 7.8 million diagnoses within the past 5 years. Conventional chemotherapy is implemented as the fundamental treatment modality for breast cancer, but the efficacy is limited due to the occurrence of multidrug resistance (MDR) [2]. Despite the advances in current treatment strategies (chemotherapy, radiotherapy, surgery, and adjuvant chemotherapy), approximately 0.69 million deaths out of 2.3 million diagnoses were reported in 2020 [3]. The dysregulation of apoptosis has been recognized as a central aspect that empowers the development of MDR. Therefore, the induction of apoptosis can be used to produce therapeutic vulnerability in cancer cells to produce breast cancer therapy.

Apoptosis (type I cell death), a mechanism designated as regulated cell death, contributes to the fine balance between cell division and death which is crucial for tissue homeostasis and the eradication of unwanted cells. The solid control in apoptosis is evident via three major groups of proteins: the inhibitors of apoptosis proteins (IAPs), Bcl-2 family proteins, and a family of caspases. The best-characterized IAPs participating in apoptotic signaling are X-linked inhibitors of apoptosis protein (XIAP), cellular IAP 1 and 2 (cIAP1/2), and Survivin. XIAP inhibits caspases by direct physical interaction [4] with its baculovirus IAP repeats (BIRs): BIR2 (caspase-3 and -7) and BIR3 domains (caspase-9). cIAP1 and cIAP2 bind to caspases-3 and -7, but they subject them toward proteasomal degradation (E3 ubiquitin ligase) through C-terminal RING finger domains instead of physical interaction and inhibition [5]. Concerning the caspase family, they induce apoptosis by activating the mitochondrial/intrinsic apoptotic pathway or by committing to the extrinsic/transmembrane apoptotic pathway [6,7]. Later, both pathways assemble at the initiator caspase level initiated by caspase-3 as feedback to stimuli from both pathways [8]. The guardian of the genome, p53 is a tumor suppressor that triggers caspase activity by the mitochondrial release of cytochrome c, but its activity is sternly modulated by a protein downstream of XIAP, MDM2, which directly interacts with the N-terminal domain of p53, initiating its degradation [9,10,11]. Therefore, MDM2 is viewed as an oncogene that has been consistently observed when overexpressed, and the modulation of the expressions of these druggable targets, along with their downstream signaling pathways, remain efficient anticancer protocols [12,13]. Based on the concept that mature caspase activity is abrogated by IAPs, their activity is intrinsically antagonized by secondary mitochondria-derived activator of caspases (SMAC), an endogenous IAP inhibitor. Therefore, an azomethine small molecule derivative, 1-(4-chlorophenyl)-*N*-(4-ethoxyphenyl)methanimine (**BCS3**) was developed and evaluated against breast cancer cells that have been reported to overexpress IAPs. Azomethines have been reported to possess a wide array of biological activities because of the imine bond (-C=N-), including cytotoxic potential in lung (A549) and cervical (HeLa) cancer cell lines [14].

The present-day investigation in the field of high-throughput breast cancer management has introduced potential biomarkers to enable the diagnosis of early-stage disease and an accurate prognosis for precision medicine and targeted therapy [15]. Golestan et al. [16] reported the upregulation of four critical genes, i.e., *CACNG4, PKMYT1, CHRNAG,* and *EPYC* that can impact prognostic significance and advancement in diagnostic and therapeutic methods. Furthermore, Lehmann et al. [17] reported the role of single nucleotide polymorphisms (SNP) in the abnormal modulation of miRNA expression in breast cancer. Therefore, the impact of SNPs on miRNA can be exploited to develop insightful breast cancer diagnostic markers. In a study reported by Bilal et al. [18], a competition-based multidimensional model was developed that can improve the analysis of survival in breast cancer patients. Large data sets of clinical and genomic patient information were used to predict improved survival compared to current methodologies. Numerous small-molecule IAP inhibitors (such as Birinapant, Tolinapant, Xevinpant, etc.) have been identified or synthesized, demonstrating their potential value as scaffolds for anti-cancer therapies [19]. Many such molecules show variations in their metabolic routes and pharmacokinetics, showing excretion through bile and entering enterohepatic recirculation, thereby increasing drug exposure [20]. Despite their clinical efficacy in advanced cancer patients, these agents induce severe hematological toxicities (thrombocytopenia, leukopenia, anemia, neutropenia) and other adverse effects that impose burdens and challenges to the healthcare system. Therefore, more multitargeted small-molecule therapeutic agents are demanded that possess maximum efficacy and good oral bioavailability with the least toxicity. Based on the above literature, the search for new synthetic motifs as potential cytotoxic and pro-apoptotic agents has identified azomethine-containing scaffolds (Figure S1) as an essential domain, as reported by previous studies [21,22]. Motivated by these findings, we evaluated the apoptotic induction efficiency of **BCS3** through the direct inhibition of IAPs and the modulation of MDM2-p53-caspases and p53-p21-CDK1/cyclin B1 axes both in vitro and in vivo to enhance tumor sensitization.

## 2. Results and Discussion

### 2.1. Chemistry

The compound **BCS3** was synthesized as per scheme I (Figure S1). **BCS3** was produced at high yield by condensing 4-chlorobenzaldehyde with 4-ethoxyaniline in the presence of glacial acetic acid as a catalyst and ethanol as a solvent. The product was characterized with FTIR, ^1^HNMR, ^13^CNMR, and mass spectrometry analyses. **BCS3** exhibited C=N stretch at 1622.02 cm^−1^, R-Cl stretch at 777.26 cm^−1^, Ar-H at 692.40 cm^−1^, and -OCH_2_CH_3_ (ketone) stretch at 1718/1733 cm^−1^. The ^1^HNMR spectra showed triplet at *δ* 1.382–1.41 and quartet at 3.992–4.05 for ketone (-OCH_2_CH_3_), *δ* 6.882–7.80 for aromatic protons, and *δ* 8.41 for C-H proton. ^13^CNMR exhibited chemical shifts at *δ* 115.05–156.69 for aromatic carbons, *δ* 63.6 for -CH_2_ (aliphatic), *δ* 14.95 for -CH_3_ (aliphatic), and *δ* 157.94 for C=N (imine) carbons. In mass spectra, a molecular ion peak was observed at 260.04 (molecular formula C_15_H_4_ClNO). The characterization results confirmed the formation of **BCS3**. The mass, ^1^HNMR, ^13^CNMR, and IR spectra for **BCS3** are given in Figures S2 and S3. The compound was found to be >95% pure by the reverse-phase HPLC method, provided in Figure S4.

### 2.2. Interpretation of Binding Patterns of BCS3 with IAPs Through In Silico Molecular Docking Studies

**Figure 1:**
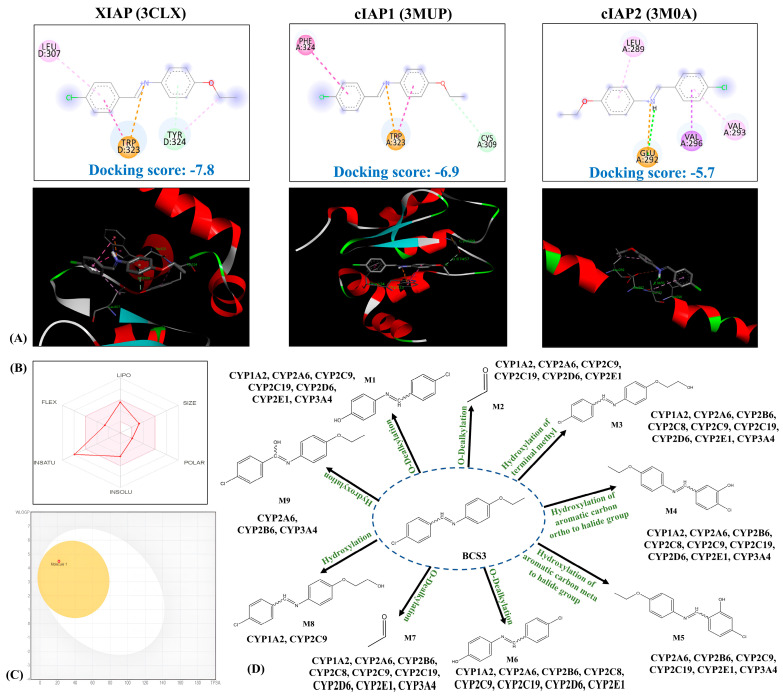
The in silico molecular docking studies of BCS3 with IAPs that represent (**A**) 2D and 3D docked poses of **BCS3** with inhibitors of apoptosis proteins (XIAP (PDB ID: 3CLX), cIAP1 (PDB ID: 3MUP) and cIAP2 (PDB ID: 3M0A). In silico ADME prediction representing (**B**) BOILED-Egg model of **BCS3** (**C**) SwissADME bioavailability radar report of **BCS3** and (**D**) Prediction of possible metabolites of **BCS3** by Phase I biotransformation.

Four azomethine derivatives (BCS1, 2, 3, and 4) were designed, and they were subjected to molecular docking using AutoDock 4.0 to predict its potential binding modes with crystal structures of IAP proteins: XIAP (PDB ID: 3CLX), cIAP1 (PDB ID: 3MUP), and cIAP2 (PDB ID: 3M0A). For the investigation of an experimentally observed ligand binding ability of molecular docking, the respective co-crystallized ligands, S22 (3CLX) and SMK (3MUP), were used as reference ligands. They were docked back into the respective binding sites of the crystal structures of XIAP and cIAP1 using AutoDock 4.0 that closely resembled the co-crystallized conformation with RMSD of 1.179 Å for XIAP, and 0.9489 Å for cIAP1 of non-hydrogen atomic positions of ligands X22 and SMK (Figure S5). This demonstrates the docking software’s capability to predict ligand binding interactions with IAP proteins accurately. Amongst the four compounds, BCS3 showed the highest binding affinity toward IAPs (Table S1). Therefore, BCS3 was chosen as the lead molecule for further studies. Table 1 presents the binding free energies (ΔG) of **BCS3** with the respective target proteins, along with details of the interacting amino acid residues. Figure 1A displays the 2D and 3D binding modes of interactions of **BCS3**. The Ala1-Val2-Pro3-Ile4 (AVPI) are four N-terminal Smac residues interacting with XIAP and cIAP1-BIR3 binding pockets. As per the study reported by Lim et al. [23], extensive interactions of potential IAP antagonists with Trp323 and Tyr324 residues initiate efficient ligand binding. The results from the molecular docking analysis revealed the capability of **BCS3** to recognize the surface pockets of IAPs (BIR3-XIAP domain) proteins and bind with good binding energies to expedite apoptosis.

### 2.3. In Silico Physicochemical Properties of BCS3

The rule of five proposed by Lipinski [24] was predicted by the Molinspiration server to investigate the drug likeness of BCS3, i.e., molecular oral bioavailability [25]. BCS3 did not violate any of Lipinski’s rules of five. BCS3 had a molecular weight of 254.74 (<500), TPSA values of 21.60 Å (<140 Å), 4 rotatable bonds (<10), higher Log *p* value, 4.58 (<5), and the number of hydrogen bonds acceptors and donors were in acceptable range (<5 and <10 respectively). The predicted values indicated good intestinal absorption, BBB permeability, good conformational flexibility, and oral activity.

The prediction of pharmacokinetic properties of BCS3 for the determination of oral compatibility, cell permeation, metabolism, elimination, and toxicity through in silico ADMET studies was performed to prevent failure in efficacy during drug development [26]. The findings suggested that BCS3 is highly likely to have good oral bioavailability and possess drug-like potential.

### 2.4. In Silico ADMET Properties of BCS3

The ADMET prediction of BCS3 was performed using in silico property prediction web servers, and the results are tabulated in Table 2. The permeability through the intestinal barrier and the uptake efficiency of BCS3 was predicted through Caco-2 cell permeability, human intestinal absorption (HIA) (showing the ability of assimilation of the drug through the intestine), and Madin-Darby canine kidney (MDCK) descriptors [27]. Figure 1B depicts the bioavailability radar that provides a graphical visualization of the drug-likeness properties of BCS3 along with the BOILED-Egg model (Figure 1C) that predicted BCS3 to be a P-glycoprotein (P-gp) inhibitor i.e., less likely to get effluxed out of the cell.

The effect of BCS3 on liver metabolizing enzymes was predicted on the major cytochrome P450 isozymes group (CYP 1A1, 3A4, 2C9, 2C19, and 2D6) that are majorly involved in the biotransformation of drugs, steroids, fatty acids, bile acids, and carcinogens [28]. As shown in Table 2(d), BCS3 was predicted to be a non-substrate and non-inhibitor of CYP enzymes. Further, the in silico metabolic study was implemented to predict the transforming enzymes, expected metabolites, and metabolic pathways likely to participate in BCS3 metabolism. The results obtained from phase I prediction of BCS3 biotransformation revealed the formation of nine possible metabolites catalyzed by cytochrome P450 isoenzymes (Figure 1D). More details are provided in the Supporting Information (Figure S6). Acute toxicity was predicted for BCS3 on specific organ toxicity (Table 2). BCS3 was found to be non-toxic in terms of AMES toxicity, hepatotoxicity, carcinogenicity, and mutagenicity. The hERG (human Ether-a-go-go-Related Gene) human model inhibition was evaluated to observe the effect of BCS3 on human toxicity. BCS3 did not show any risks for hERG inhibition [29].

### 2.5. Biological Studies

#### 2.5.1. Cell Viability Analysis on Breast Cancer Cell Lines

**Figure 2:**
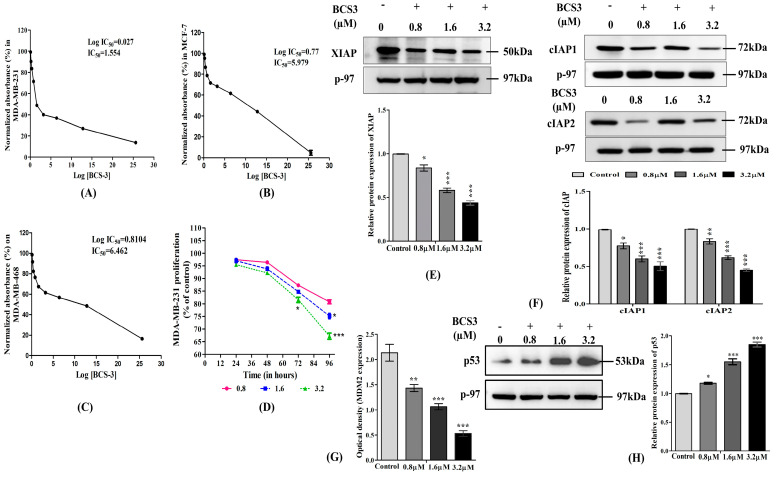
Cytotoxic effects of various concentrations of **BCS3** on the proliferation of breast cancer cells, (**A**) MDA-MB-231, (**B**) MCF-7, and (**C**) MDA-MB-468 through MTT assay for 24 h. (**D**) Time-course study of proliferation of MDA-MB-231 cells after incubation with varying doses of **BCS3** (0.8, 1.6, and 3.2 µM) up to 96 h. Cell viability of the DMSO vehicle control group was set at 100%. Effect of varying doses of BCS3 (0.8, 1.6, and 3.2 µM) on the relative protein expressions of inhibitory apoptotic proteins (IAPs) as quantified by western blotting analysis in MDA-MB-231 cells (**E**) XIAP, (**F**) cIAP1, and cIAP2, respectively. Respective graphs represent each band of cIAP1 and cIAP2 measured by densitometry and normalized to corresponding p-97. (**G**) Protein analysis of MDM2 by ELISA in MDA-MB-231 cells. (**H**) Relative protein expression of p53 quantified by western blotting in MDA-MB-231 cells. The respective graph represents each band of XIAP, cIAP1, cIAP2, and p53 measured by densitometry and normalized to corresponding p-97. Mean ± SEM was calculated by replicating the experiment thrice (n = 3). Significant differences are shown as * *p* < 0.05, ** *p* < 0.01, *** *p* < 0.001, with the control group.

**BCS3** was subjected to cytotoxicity screening using MTT assay against various breast cancer cell lines, MCF-7 (Figure 2A), MDA-MB-231 (Figure 2B), and MDA-MB-468 (Figure 2C) for evaluation of anti-breast cancer potential. The IC_50_ value of **BCS3** was predicted with serial dilutions in each cell line, where **BCS3** was found to be most potent against MDA-MB-231 cells (hormone negative). **BCS3** exhibited remarkable inhibitory potential against the MDA-MB-231 cell line (IC_50_ value = 1.554 µM), followed by MCF-7 (IC_50_ value = 5.979 µM) and MDA-MB-468 (IC_50_ value = 6.462 µM). The cytotoxic selectivity of **BCS3** toward breast cancer cells was screened by cell viability assay on MCF-10A cells (non-tumorigenic breast epithelial cells) which was found to be IC_50_ = 69.62 µM, reflecting higher affinity against cancer cells.

Since **BCS3** showed 10 times more selectivity toward MDA-MB-231 cells, it was subjected to a time-course study for 96 h where cell viability was observed to have declined at 1.6 and 3.8 µM concentration post-incubation of 48 h (Figure 2D). A significant reduction (*p* < 0.5) in viability was observed between lower (0.8 µM) and higher (3.8 µM) doses after 72 h of treatment with **BCS3**. The effect of the cell viability of **BCS3** was tested against various breast cancer cell lines (MCF-7, MDA-MB-231, and MDA-MB-486), amongst which maximum potency was observed in MDA-MB-231 cells (IC_50_ = 1.554 µM) in a dose and time-dependent manner. **BCS3** also showed selective inhibition toward other breast cancer cells (MCF-7 and MDA-MB-486) without affecting normal breast epithelial cells, MCF-10A (IC_50_ > 50 µM) thus depicting its low toxicity [30].

#### 2.5.2. BCS3 Inhibited the Expression of IAP and Downstream Proteins in MDA-MB-231 Cells

The maximum potency of **BCS3** was observed in MDA-MB-231 cells, therefore, further biological analyses to confirm the cell death mechanistic approach of **BCS3** were conducted on MDA-MB-231 cells. For the determination of the participation of IAPs in the apoptotic mechanism, the levels of IAPs, XIAP (Figure 2E), cIAP1, and cIAP2 (Figure 2F) were accessed by the western blotting technique. The expression of IAPs in MDA-MB-231 cells was much higher, but upon **BCS3** treatment, subsequent proteasomal depletion of IAPs was observed by stimulation of autoubiquitination [31] dose-dependently. Next, the molecular mechanism of MDM2 and p53 regulation by XIAP was investigated by ELISA and immunoblotting, respectively. The depletion of XIAP caused a significant decline in MDM2 expression (Figure 2G) which accelerated the accumulation of tumor suppressor protein, p53 [32] dose-dependently (Figure 2H).

Further in vitro biological evaluation was conducted on MDA-MB-231 cells, which are categorized as highly aggressive, metastatic, and progressive forms of breast cancer with limited systemic treatment options [33]. In this study, the antagonism of IAPs by **BCS3** resulted in the prompting of multiple downstream mechanisms, i.e., IAP-MDM2-mediated p53 accumulation that caused the off-target regulation of downstream pro- and anti-apoptotic, IAP-inhibition-mediated direct caspase activation causing intrinsic and extrinsic apoptosis and p53-dependent p21-CDK1/cyclin B1-mediated cell cycle arrest at S and G2/M phases leading to tumor growth inhibition in breast cancer cells.

#### 2.5.3. BCS3-Triggered Intrinsic/Mitochondrial Apoptosis in MDA-MB-231 Cells

**Figure 3:**
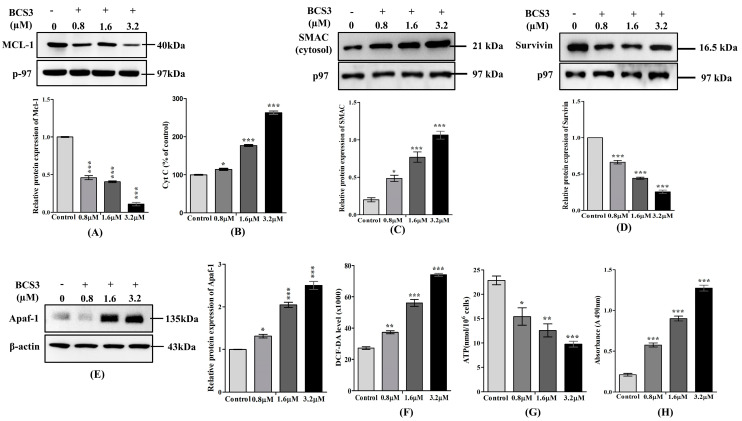
Relative protein expression of (**A**) MCL-1 quantified by western blotting in MDA-MB-231 cells. (**B**) Protein analysis of cytochrome c by ELISA in MDA-MB-231 cells. Relative protein expression of (**C**) SMAC, (**D**) Survivin, and (**E**) Apaf-1 quantified by western blotting in MDA-MB-231 cells. The respective graph represents each band of MCL-1, SMAC, Survivin, and Apaf-1 measured by densitometry and normalized to corresponding p-97 and β-actin. Cytotoxicity assessment through measurement of (**F**) ROS, (**G**) ATP content, and (**H**) lactate dehydrogenase (LDH) activity in MDA-MB-231 cells for 24 h post-treatment with varying doses of BCS3 (0.8, 1.6, and 3.2 µM). Mean ± SEM was calculated by replicating the experiment thrice (n = 3). Significant differences are shown as * *p* < 0.05; ** *p* < 0.01 and *** *p* < 0.001with the control group.

The profound role of p53 in apoptosis has been extensively reported. p53 activation by **BCS3**-mediated IAP inhibition downregulated the expression of anti-apoptotic protein, MCL-1 as quantified by western blotting (Figure 3A). The cytosolic release of cytochrome c was examined by ELISA in **BCS3**-treated MDA-MB-231 cells which showed a significant (*p* < 0.001) elevation with a gradual rise in drug dose (Figure 3B). Upon induction of apoptotic stimuli, SMAC, a negative IAP regulator was also released into the cytosol alongside cytochrome c which was quantified by immunoblotting as depicted in Figure 3C. The release of SMAC downregulated the IAPs including Survivin by promoting their ubiquitination to initiate apoptosis [34] as quantified by immunoblotting (Figure 3D). The released cytochrome c in the cytosol interacted with apoptosis-protease activating factor-1 (Apaf-1), forming a complex, Apoptosome (multi-protein seven-spoked ring-shaped complex) that initiated the caspase cascade. The elevated level of Apaf-1 after **BCS3** treatment examined through immunoblotting (Figure 3E) confirmed the formation of a complex [35].

**Figure 4:**
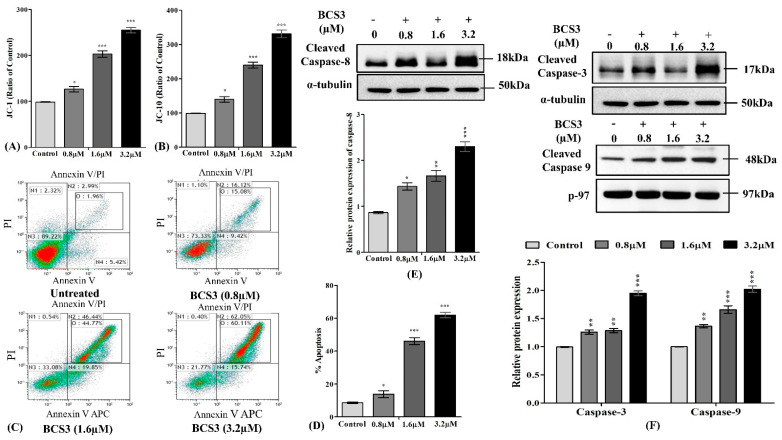
Analysis of mitochondrial membrane potential of BCS3 on MDA-MB-231 cells verified using (**A**) JC-1 and (**B**) JC-10 levels. Mean ± SEM was calculated by replicating the experiment thrice (n = 3). Significant differences are shown as * *p* < 0.05; ** *p* < 0.01 and *** *p* < 0.001 with the control group. (**C**) Determination of apoptotic cell death in MDA-MB-231 cells documented by annexin V-Alexa Fluor 647 (a647)/PI double staining and FACS analysis after treatment with BCS3 (0.8, 1.6, and 3.2 µM). Annexin V[+] and PI[−]: apoptotic cells (N4); annexin V[+] and PI[+]: Late apoptotic cells (N2); annexin V[−] and PI[+]: necrotic cells (N1) and annexin V[−] and PI[−]: Living cells (N3). (**D**) Bar diagram depicting the percentage of apoptosis caused by a number of early and late apoptotic cellular populations after BCS3 treatment. Representation of western blot analysis showing the protein expression of (**E**) cleaved caspase-8, (**F**) cleaved caspase-3, and cleaved caspase-9 after 72 h of treatment with BCS3 (0.8, 1.6, and 3.2 µM). Respective graphs represent each band of caspases (3 and 9) measured by densitometry and normalized to corresponding α-tubulin and p-97. Mean ± SEM was calculated by replicating the experiment thrice (n = 3). Significant differences are shown as * *p* < 0.05; ** *p* < 0.01 and *** *p* < 0.001 with a control group.

To quantify the extent of DNA damage caused by **BCS3**, the intracellular ROS level was examined (Figure 3F) along with an evaluation of the ATP level (Figure 3G). **BCS3**-treated groups depicted an upsurge in the generation of intracellular ROS and ATP depletion content dose-dependently that resulted in mitochondrial dysfunction [36]. The extent of cellular damage was also measured by the LDH enzymatic level using LDH assay. Untreated MDA-MB-231 cells showed the least LDH release when examined through an absorbance value of 0.211. LDH release significantly (*p* < 0.001) elevated post-treatment with **BCS3** with increasing doses (Figure 3H) which was indicative of cell death caused by the destruction of cellular membrane and apoptosis [37]. MMP was quantified by measuring the fluorescent intensities for both the monomeric form and the J-aggregates of JC-1 and JC-10. The untreated MDA-MB-231 cells showed a lower intensity of fluorescence which enhanced significantly after **BCS3** treatment that stipulated the induction of apoptosis (Figure 4A,B).

#### 2.5.4. BCS3 Initiated Apoptosis in MDA-MB-231 Cells Through Initiation of Caspase Cascade

After a mechanistic analyses of apoptotic proteins, the mediation of apoptosis by BCS3 was investigated by flow cytometry (Figure 4C,D). MDA-MB-231 cellular distribution was observed in four quadrants (N1, N2, N3, and N4) post-treatment with annexin V-alexa-fluor 647 and PI stain that acts as apoptosis indicator. Untreated MDA-MB-231 cells showed maximum cellular distribution in Q3 (annexin V[−] and PI[−]). Treatment with various concentrations of BCS3 (0.8, 1.6, and 3.2 µM) caused a noticeable elevation in cellular distribution in annexin V[+] and PI[−] for initial apoptotic cells in MDA-MB-231 cells followed by the expansion of late-apoptotic cell distribution (annexin[+] and PI[+]) as compared to untreated cells. The overall percentage of apoptosis elevated significantly after BCS3 treatment dose-dependently.

**Figure 5:**
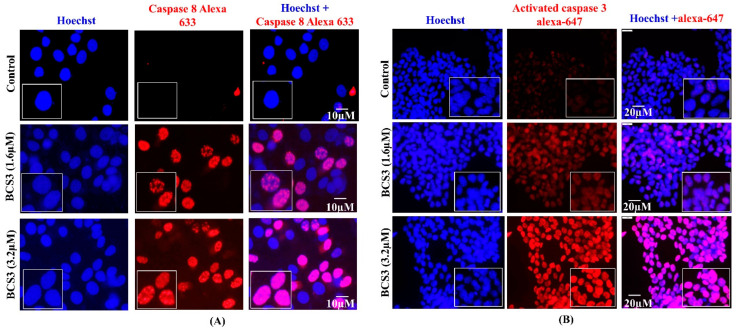
Immunofluorescence analysis depicting the elevation of (**A**) cleaved caspase-8 and (**B**) cleaved caspase-3 in vitro in MDA-MB-231 cells after 72 h of treatment with varying doses of BCS3 (1.6 and 3.2 µM) where Alexa 633 (red fluorescence) denotes caspase-3 and -8 expressions, respectively, and Hoechst (blue fluorescence) denotes cell nuclei locations. The scale bars of immunofluorescence analysis are represented as 10 µm.

The final outcome which executes apoptosis is the potentiation of proteases termed caspases. The extrinsic (caspase-8) and intrinsic (caspase-9) apoptotic pathways converge at caspase-3 activation which acts as a final signal for the accomplishment of apoptosis. The cleavage of caspases within their proteolytic domains results in their activation producing a larger and smaller subunit [38]. Based on enhanced apoptosis in MDA-MB-231 cells examined through flow cytometry, the next aim was to quantify caspase involvement through western blotting and immunofluorescence analysis. The expressions of cleaved caspase-8 (Figure 4E), cleaved caspase-3, and cleaved caspase-9 (Figure 4F) were notably enhanced after BCS3 treatment dose-dependently as depicted by western blotting analysis. The protein expression of caspases was further assisted and validated by immunofluorescence analysis. The expressions of caspase-8 (Figure 5A) and caspase-3 (Figure 5B) were notably enhanced after BCS3 treatment as marked with the expansion of red fluorescence by Alexa Fluor 647.

The apoptotic pathways are strictly regulated by IAPs; therefore, overexpressed IAPs help the cancer cells evade apoptosis. The antagonism of IAPs (XIAP and cIAP1/2) by BCS3 was confirmed by western blot analysis in MDA-MB-231 cells. This could be due to the induction of the proteasomal degradation of cIAP proteins. cIAPs promote receptor-interacting protein 1 (RIP1) release from the TNFR1 complex resulting in RIP1-dependent caspase-8 activation [39,40]. IAP inhibition by BCS3 mediated downregulation of downstream protein, MDM2 which resultantly disrupted its interaction with p53 to induce cell senescence [41] as observed by ELISA and western blot analyses. p53 upregulation induced the direct association and proteasomal degradation of overexpressed Bcl-2 family protein, Mcl-1, in cytoplasm, thereby promoting apoptosis induction [42]. Aside from Bcl-2 proteins, an enhanced ROS level oxidizes mitochondrial pores which releases cytochrome c from the mitochondrial intermembrane space to cytosol by collapsing of MOMP. BCS3 elevated the intracellular ROS level and depleted ATP which resulted in the enhancement of cytosolic cytochrome c level. Cytochrome c combined with Apaf-1 and pro-caspase-9 apoptosome resulted in cell death which demonstrated the activation of the mitochondrial apoptotic pathways. The release of mitochondrial proteins (cytochrome c and SMAC) into cytosol is critical for activating caspases. Despite being two independent events, previous studies have reported that the release of SMAC promotes translocation of cytochrome c in cytosol [43]. SMAC being an endogenous IAP (XIAP, cIAP1/2, and Survivin) antagonist, along with BCS3-mediated IAP inhibition, caused disruptions in interactions with initiator caspase-9 and executioner caspase-3 further promoting direct apoptosis by the displacement of caspases from XIAP binding pockets. Based on enhanced apoptosis in BCS3-treated MDA-MB-231 cells (observed by flow cytometry), the involvement of caspases was confirmed by western blotting and immunofluorescence analyses.

#### 2.5.5. BCS3-Induced S and G2/M Phases Cell Cycle Arrest by p21 Upregulation and CDK1-Cyclin B1 Downregulation in MDA-MB-231 Cells

**Figure 6:**
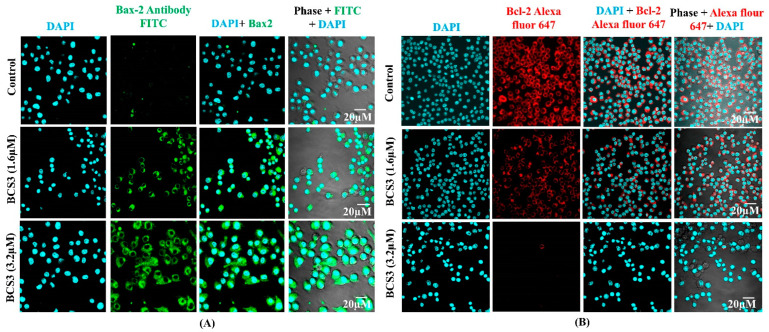
(**A**) Immunofluorescence analysis depicting the elevation of Bax in vitro in MDA-MB-231 cells after 72 h of treatment with varying doses of BCS3 (1.6 and 3.2 µM) where FITC (green fluorescence) denotes Bax expression and DAPI (light blue fluorescence) denotes cell nuclei locations. (**B**) Immunofluorescence analysis depicting the depletion of the Bcl-2 protein in vitro in MDA-MB-231 cells after 72 h of treatment with varying doses of BCS3 (1.6 and 3.2 µM) where Alexa 647 (red fluorescence) denotes Bcl-2 expression and DAPI (light blue fluorescence) denotes cell nuclei locations. The scale bars are represented as 20 µm.

The activation of p53 caused by DNA damage led to the induction of pro-apoptotic protein transcription, Bax and Bak, and the inhibition of anti-apoptotic protein transcription, Bcl-2, Bcl-xL. The expression of Bax and Bcl-2 proteins were analyzed by immunofluorescence analysis post-treatment of MDA-MB-231 cells with BCS3. The fluorescence intensity was observed to increase with the marked enhancement of the green fluorescence color of FITC dye showing upregulation of Bax (Figure 6A). Another finding of diminished red fluorescence intensity of Alexa Fluor 647 dye was observed when MDA-MB-231 cells were treated with BCS3 dose-dependently which caused the marked downregulation of Bcl-2 protein expression (Figure 6B) causing cellular apoptosis.

**Figure 7:**
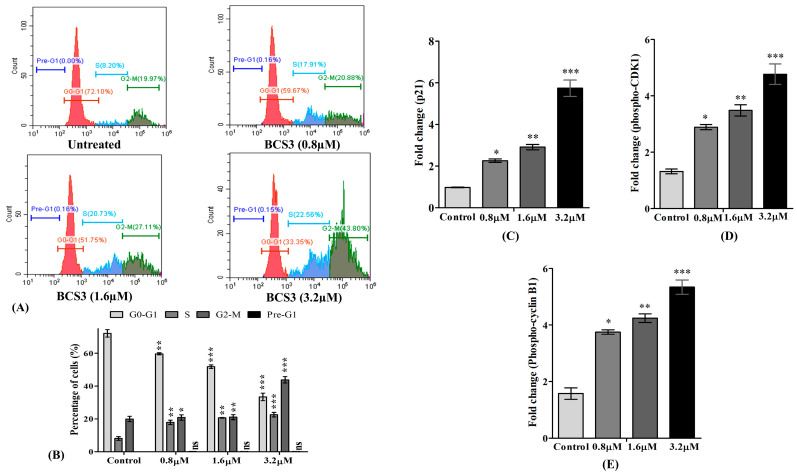
Flow cytometric analysis indicating (**A**) cell cycle progression of untreated MDA-MB-231 cells, the effect of different concentrations of BCS3 (0.8 µM, 1.6 µM and 3.2 µM) on MDA-MB-231 cells, (**B**) percentage of cell cycle distribution protein analysis of (**C**) p21, (**D**) phospho-CDK1, and (**E**) phospho-cyclin B1 by ELISA. Mean ± SEM was calculated through replicating the experiment thrice (n = 3). Significant differences are shown as * *p* < 0.05; ** *p* < 0.01 and *** *p* < 0.001 with the control group.

Based on previous reports of cell cycle interruption by IAP inhibitors [44], the effect of BCS3 to hamper the cell cycle of tumor cells was examined by flow cytometry. The content of cellular DNA in dividing cells (Figure 7A) was estimated by PI staining along with cell population through cell cycle arrest caused by S and G2-M phases. Three different concentrations of BCS3 (0.8, 1.6, and 3.2 µM) were used to treat MDA-MB-231 cells. Post-treatment with gradual concentrations of BCS3, the cell percentage of the phases in the cell cycle was observed as shown in Figure 7B. For the further evaluation of cell cycle arrest at the S and G2-M phases, the expression of cell cycle regulatory proteins was quantified. Due to the presence of two conserved p53 binding sites in the p21 protein that respond to DNA damage, the p21 expression was quantified through ELISA (Figure 7C). An upregulation of p21 expression was noted with gradually increasing doses of BCS3. The p21, being a negative regulator of the cell cycle at G1/S and G2/M transition, plays a vital role in tumorigenesis inhibition by blocking the kinase activity of CDK1/cyclin B1, arresting growth at the G2/M phase [38,44]. The phospho-CDK1 and phospho-cyclin B1 levels of MDA-MB-231 cells were also elevated by BCS3 dose-dependently that implied protein deactivation [45] (Figure 7D,E).

Apoptosis and cell cycle deregulation are thinly connected events, and disruption in cell cycle progress ultimately results in apoptotic death [46]. Therefore, the cell percentage of apoptosis in MDA-MB-231 cells was quantified by FACS analysis after BCS3 treatment, and the findings supported the cell death event. The high accuracy of the technique is achieved by determining the DNA content within the cell by the use of fluorescent dye, propidium iodide (PI), which binds to genetic material (mitochondrial DNA, DNA, and RNA) after the rupture of the cell membrane by cytotoxic drugs [47]. Additionally, the cell cycle analysis projected toward cell cycle termination at the S phase and the G2/M phase when MDA-MB-231 cells were treated with BCS3. p53 activation by IAP inhibition activated downstream protein, p21, which is a prime regulator of CDK1 and cyclin B1 proteins. The resultant complex of CDK1/cyclin B1 (cyclins activate CDKs to initiate cell cycle progression) is specified as a G2/M phase regulator. p53 has a crucial role in regulating genome integrity and DNA repair by controlling checkpoints during cell division. In the cancer state, the G2/M phase relies on checkpoint kinases, which are highly disrupted, causing infinite proliferation. The mRNA expression levels of IAPs, Bcl-2 family members, caspases, and cell cycle proteins were quantified using the RT-qPCR technique before and after treating MDA-MB-231 cells with BCS3, where the successful restoration of altered levels was observed (Figure S7). All the results were consistent with the dose-dependent effects of BCS3. This could be possibly due to blocking the protein–RNA interaction of IAPs (XIAP, cIAP1/2) that can degenerate the protein [48].

#### 2.5.6. BCS3 Reduced Tumor Volume and Restored Alterations in Body Weight and Cellular Architecture in Cancer-Bearing Animals

**Figure 8:**
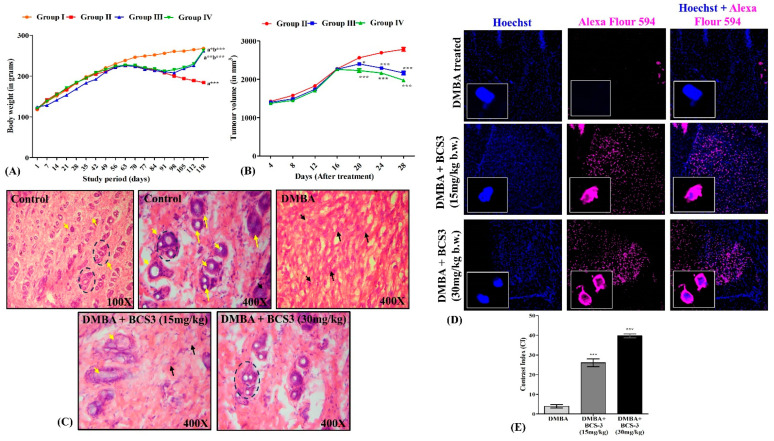
(**A**) Effect of BCS3 on body weight (in grams) of DMBA-induced breast cancer in experimental rats. (**B**) Effect of BCS3 on tumor volume of DMBA-induced breast cancer in experimental rats. (**C**) Histopathological observation (400×) of hematoxylin and eosin (H&E)-stained mammary tissues where scale bare represents the 50 µm section. Yellow arrows represent normal acini and ductules in normal groups. Black arrows show ductal hyperplasia in DMBA-treated group with abnormal tissue architecture. BCS3-treated groups show restoration of ductal architecture by yellow arrows. (**D**) Representation of TUNEL fluorescent images of a section of tumor excised from experimental animals from each group. Cell nuclei locations are indicated with Hoechst (blue fluorescence) and apoptotic cell location is indicated by Alexa Fluor 594 (purple fluorescence). The scale bars of immunofluorescence analysis are represented as 100 µm. (**E**) Evaluation of the rate of tumor cell apoptosis in mammary tissues of rats from each experimental group in accordance with images of TUNEL assay. Comparisons: a-Groups II, III, and IV with Group I; b-Groups III and IV compared to Group II; *** *p* < 0.001, ** *p* < 0.01 and * *p* < 0.05. Group I: Control; Group II: induced control (DMBA, 20 mg in 0.5 mL of olive oil); Group III: DMBA (20 mg) + BCS3 (15 mg/kg, b.w); and Group IV: DMBA (20 mg) + BCS3 (30 mg/kg, b.w). DMBA: 7,12-dimethylbenz(a)anthracene.

The transition of anti-breast cancer potential of BCS3 was evaluated in vivo in DMBA-treated female SD rats. Since the anatomical parameters directly correlate with physiological abnormalities, the body weight, tumor volume, and histopathological analyses were recorded to quantify the anti-cancer effect of BCS3. As the control group showed a steady and gradual increase in body weight, the DMBA-treated animals were noted with an accelerated decline in body weight after the 11th week of the study protocol. It could be a result of loss in body mass due to cancer cachexia, anorexia, and muscle weakness caused by impaired metabolism and malabsorption [49]. Remarkable improvement was noted in animal body weight upon treatment with BCS3 dose-dependently compared to the induced control group, which showed maximum protection and efficacy (Figure 8A). Subsequently, noticeable mammary tumors were observed after 14 days of the 20 mg DMBA treatment, followed by 90 days of the tumor promotion phase [50]. The tumor promotion phase was followed by a 28-day treatment period in treatment groups where significant and potent regression in tumor volume was observed post-BCS3 treatment dose-dependently, further validating its anti-tumor potential (Figure 8B).

The analysis of changes in breast/tumor tissue cellular architecture before and after treatment with BCS3 and histopathological evaluation was performed. A mature and normal mammary gland in the normal control group exposits uniformly arranged lactiferous ducts, which are surrounded by well-differentiated single-layered epithelial cells. The ductules and acini present show normal architecture (yellow arrows). The mammary tumor tissue (DMBA-treated group) obtained from cancer-bearing animals portrayed deformed structures with a hyper-chromatinized nucleus showing characteristics of ductal adenocarcinoma caused by epithelial proliferation (black arrows). BCS3 treatment (15 mg/kg) presented marked improvement in the deformed architecture of breast tissue and ductules. Yellow and black arrows show improved mammary tissue architecture. Treatment with a higher dose of BCS3 (30 mg/kg b.w) showed a marked reduction in hyper-chromatinization and hypertrophy of cells and a restoration of the deformities of epithelial cell arrangement that was caused by fibroadenoma. The encircled parts show mammary ducts with improved epithelial alignments (Figure 8C).

Consistent with its role, BCS3 showed potent cytotoxic and antiproliferative activities in an in vivo breast cancer model in SD rats. Rat mammary carcinogenesis serves as an efficient tool, as it provides analogous relation to human mammary carcinogenesis [51]. The restoration of body weight, reduction in tumor volume, and restoration of cellular architecture by histopathological evaluation validated the in vivo anti-cancer potential of BCS3.

#### 2.5.7. BCS3 Triggered Induction of Apoptosis in Cancer-Bearing Animals

To evaluate the in vivo apoptosis-inducing potential of BCS3, tumors excised from experimental animals were subjected to a TUNEL assay to examine the apoptotic rate (Figure 8D,E). The purple fluorescence signals obtained from the tissues of DMBA-treated animals showed minimum visibility of Alexa Fluor 594. Upon treatment with consecutive BCS3 doses, advanced apoptosis was marked which was captured with a significant elevation of Alexa Fluor 594 fluorescence intensity. Therefore, the varying extent of TUNEL-positive cells captured the occurrence of intra-tumoral apoptosis by BCS3. Furthermore, the mechanistic involvement of major apoptotic proteins was quantified by analyzing the in vivo expressions of Bax, cleaved caspase-3, XIAP, and cell cycle proteins, p21, phospho-CDK1, and phospho-cyclin B1.

**Figure 9:**
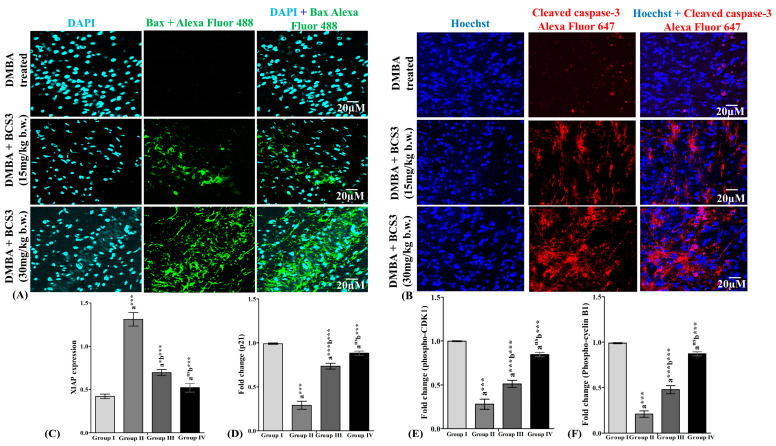
(**A**) Immunofluorescence analysis of Bax in mammary tissues belonging to cancer-bearing animals. Expression of Bax was observed to enhance upon BCS3 treatment which was observed by Alexa Fluor 488 (green fluorescence) representing Bax expression and DAPI (light-blue fluorescence) denoting cell nuclei locations. The scale bars of immunofluorescence analysis are represented as 20 µm. (**B**) Immunofluorescence analysis of cleaved caspase-3 in mammary tissues belonging to cancer-bearing animals. Expression of cleaved caspase-3 was observed to enhance upon BCS3 treatment which was observed by Alexa Fluor 647 (red fluorescence) representing cleaved caspase-3 expression and Hoechst (blue fluorescence) denoting cell nuclei locations. The scale bars of immunofluorescence analysis are represented as 20 µm. (**C**) Effect of BCS3 on protein expression of XIAP. Effect of BCS3 on expression of cell cycle proteins (**D**) p21, (**E**) phospho-CDK1, and (**F**) phospho-cyclin B1 on mammary tissues of experimental animals. Comparisons: a-Groups II, III, and IV with Group I; b-Groups III and IV compared to Group II; *** *p* < 0.001, * *p* < 0.05, and ^ns^ *p* > 0.05. Group I: Control; Group II: induced control (DMBA, 20 mg in 0.5 mL of olive oil); Group III: DMBA (20 mg) + BCS3 (15 mg/kg, b.w); and Group IV: DMBA (20 mg) + BCS3 (30 mg/kg, b.w). DMBA: 7,12-dimethylbenz(a)anthracene.

The protein expressions of Bax and cleaved caspase-3 were examined through immunofluorescence analysis on mammary tissues excised from experimental animals. DMBA-treated animals showed observably diminished levels of pro-apoptotic protein, Bax that elevated after BCS3 treatment dose-dependently as observed through green colored fluorescence (Alexa Fluor 488) (Figure 9A). Further, cytoplasmic localization of cleaved caspase-3 was observed to have reduced with the induction of mammary carcinogenesis. After BCS3 treatment, the expression was notably expanded with increased red color intensity (Alexa Fluor 647) (Figure 9B). The evident inhibition of XIAP expression in vivo was also observed through ELISA (Figure 9C). Then, the levels of cell cycle regulator proteins (p21, phosphor-CDK1, and phosphor-cyclin B1) were examined through ELISA (Figure 9D–F). The evident reduction in cell cycle protein expression post BCS3 treatment in a dose-dependent fashion was noted in accordance with previous reports that postulated the critical role of CDK1/cyclin B1 complex attenuation in arresting G2/M transition in cancer cells [28].

The occurrence of the apoptotic event was confirmed by TUNEL assay and the mechanism of apoptosis was quantified by examining the expressions of critical apoptotic proteins, XIAP, Bax, cleaved caspase-3, and cell cycle proteins, p21, phosphor-CDK1, and phospho-cyclin B1, which showed restoration near normalcy after the BCS3 treatment.

#### 2.5.8. The In Vitro Synergistic Effects of BCS3 and Doxorubicin Combination Through Synergyfinder 3.0

**Figure 10:**
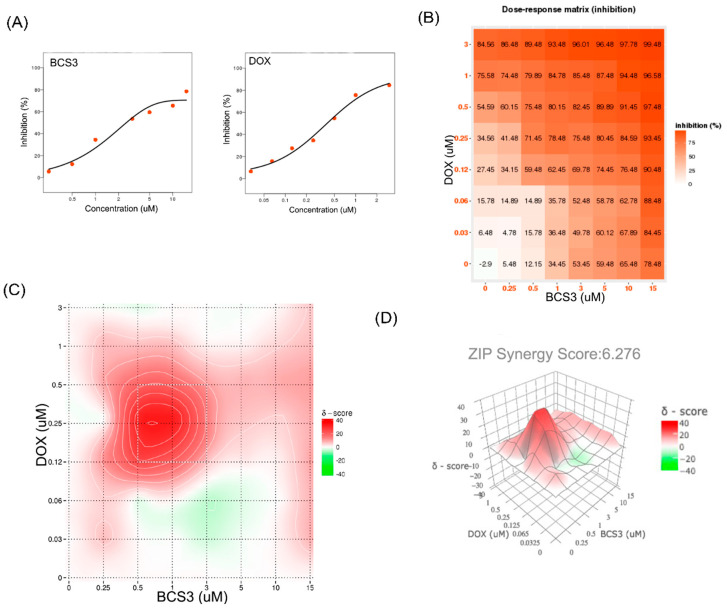
(**A**) Dose-inhibition response curve for BCS3 and doxorubicin output; (**B**) dose-response matrix (inhibition)/heat map for BCS3 and doxorubicin where the degree of red is positively related to inhibition ratio; (**C**) drug interaction landscape; and (**D**) synergy plot of combined treatment of BCS3 and doxorubicin calculated with zero interaction potency (ZIP) reference model of Synergyfinder.

To determine the combinatorial effects of BCS3 and doxorubicin in MDA-MB-231 cells, the Synergyfinder tool was used (Figure 10). The mean dose-response curves of BCS3 and doxorubicin in MDA-MB-231 cells are depicted in Figure 10A. The dose-response matrix showing the inhibition ratio in MDA-MB-231 cells depicted a positive response and the best synergistic concentration was achieved with 3 µM Dox + 15 µM BCS3 (Figure 10B). The comprehensive 2D and 3D maps over the dose matrix graphically visualized an average ZIP synergy score of 6.276 that indicated the synergistic effect of the proposed drug pair (Figure 10C,D). The ZIP model assesses the relationship between drug interactions by comparing the difference in the potency of individual drugs’ dose-response curves along with their combinations [52].

Chemotherapy remains an important treatment strategy for advanced breast cancer. Doxorubicin (also called adriamycin) is the drug of choice for breast cancer and induces cell death by intercalating with DNA, thereby blocking its synthesis [53]. Resistance to doxorubicin through apoptotic evasion is a major obstacle in its clinical application. The synergistic antitumor effects of doxorubicin with IAP inhibitors have been reported by several studies in various cancers. Therefore, the effects of BCS3 in combination with doxorubicin were identified using the ZIP reference model of Synergyfinder. The effects of the drug pair showed a synergistic relationship in MDA-MB-231 cells, which might be due to an enhanced apoptotic response by IAP inhibition by BCS3 that might have otherwise helped in cancer cell survival against doxorubicin-induced cellular stress.

#### 2.5.9. Biocompatibility Evaluation of BCS3 as Indicated by Hematological, Biochemical, and Histological Evaluation

The assessment of in vivo toxicity on normal Wistar rats orally administered with BCS3 at 30 and 300 mg/kg doses daily for 28 and 42 days (satellite groups), respectively, showed no notable toxic symptoms such as shivering, tremor, restlessness, wheezing, enhnaced salivation and respiration throughout the study period. The body weight of experimental animals of all groups showed a steady and gradual increase as recorded at 28 and 42 days as shown in Table S2. Furthermore, no visible toxic changes were noted on hematological parameters during the study period of different groups. However, liver marker enzyme levels were notably elevated upon higher dose (300 mg/kg) administration, which reverted to normalcy after discontinuation of the drug as shown in Table S3.

**Figure 11:**
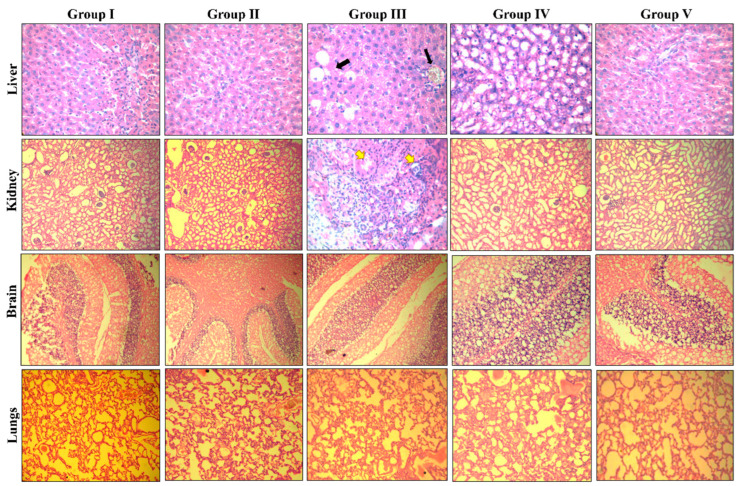
Representative photomicrograph of vital organs (liver, kidney, brain, and lungs) of normal experimental rats from repeated dose sub-acute oral toxicity study. Black arrows represent the presence of lipid droplets caused by hepatic steatosis. The kidney represents cloudy swelling (yellow arrows) and leukocytic infiltration as an effect of repeated administration of 300 mg/kg/day BCS3 dose. Group I: vehicle control group; Group II: 30 mg/kg/day of BCS3; Group III: 300 mg/kg/day of BCS3 (28 days); Group IV: satellite control group; and Group V: 300 mg/kg of BCS3 (satellite groups: 42 days); n = 5.

Histological analysis of vital organs (liver, kidney, brain, and lungs) was performed to support the biochemical finding and identification of any cellular alterations caused as an effect of repeated BCS3 treatment as shown in Figure 11. No cellular alterations were observed in the vital organs at 30 mg/kg of the BCS3 dose. However, the animals treated with 300 mg/kg repeated doses of BCS3 showed acute toxic mild hepatic damage by the development of microgoticular hepatic steatosis with the presence of lipid droplets and prominent congestion in hepatic vessels (black arrows). The kidneys of higher-dose-treated animals showed the infiltration of leukocytes along with cloudy swelling (yellow arrows). The damage caused by the toxic effects of higher doses of BCS3 on the liver and kidneys of rats reverted to normal after discontinuation of the drug (satellite group). No further toxic gross abnormalities were observed in the brain and lungs.

Preliminary toxicity assessment in normal rats following OECD 407 guidelines showed that BCS3 was well tolerated at the therapeutic dose of 30 mg/kg as examined by hematological, biochemical, and histological features. The hematopoietic system is very vulnerable to harmful substances and is very sensitive to chemotherapeutic agents. As observed by the undisturbed WBC, platelets, neutrophils, and lymphocyte counts, BCS3 did not exert any challenges on the immune and hematopoietic systems of animals. Overall toxicological evaluation findings stipulated that the therapeutic dose of BCS3 that resulted in significant tumor suppression was well tolerated in rats without apparent toxicity to normal tissues.

## 3. Materials and Methods

### 3.1. General Considerations

All chemical reagents used in this study were purchased from commercial suppliers (Sigma-Aldrich, Bangalore, India). The progress of the formation of the reaction product was checked using pre-coated TLC plates (Merck, Bangalore, India) in an 8:2 ratio of petroleum ether (40–60): ethyl acetate (solvent system). The solvent was excluded from the final products with the help of a rotary evaporator (R-100 SJ29/32, V,230V with V100 vacuum pump, Buchi Labortechnik AG, St. Gallen, Switzerland). The melting point of the synthesized product was checked using Optimelt (Stanford Research System, Sunnyvale, CA, USA). Carbon, nitrogen, hydrogen, and oxygen elemental analyses were performed using the Elemental analyzer, Hesse, Germany, Vario EL III. The characterization of final products was carried out using ESI-MS (Thermo Scientific, Waltham, MA, USA), ^13^CNMR, and ^1^HNMR using a JEOL 400 MHz instrument, Tokyo, Japan, and CDCl_3_ was used as a solvent. Tetramethylsilane was considered as an internal standard (*δ* 0.00 ppm) (^13^CNMR: *δ* 39.5 ppm of CDCl_3_; ^1^HNMR: *δ* 2.5 ppm of CDCl_3_). The infrared spectrum was obtained using Shimadzu Corpn., Kyoto, Japan, IR-Prestige 21 using KBr optics. The purity statement of BCS3 was determined by high-performance liquid chromatography (HPLC) on Thermo Scientific, USA (Vanquish) with the following conditions: 50–70% CAN in water, 20 min run, flow rate of 0.2 mL/min, UV detection (λ = 271 nm). Computational studies were carried out on a Dell precision workstation (Ryzen 5 Quad Core Processor, 8 GB RAM).

#### Synthesis of BCS3

The design and synthesis of BCS3 was performed after slight modifications in the scheme proposed by Sahu et al. (Scheme I) [54]. The details of compound synthesis and characterization are listed in the Supplementary File (Figures S1–S4).

### 3.2. Prediction of Binding Affinity Through In Silico Molecular Docking Studies

Chem Draw Ultra 10.0 [55] was used to draw the 2D and 3D structure of BCS3. The docking calculations for BCS3 were performed using the AutoDock 4.0 program [56,57]. The X-ray crystal structures of IAP proteins XIAP (PDB ID: 3CLX), cIAP1 (PDB ID: 3MUP), and cIAP2 (PDB ID: 3M0A), respectively, were used for the study and were obtained from Protein Data Bank [58]. The downloaded apoptotic protein structures were prepared using the protein preparation wizard in Swiss PDB viewer v4.1 (SPDBV) [59]. Their respective structures were optimized by the removal of water molecules, hetero atoms, and co-factors. The computation of missing atoms, hydrogen bonds, and charges was performed. A receptor grid was generated around the respective apoptotic proteins’ active sites by choosing a grid box of 9 Å. The analysis of the docking study was performed based on the docking scores and the binding modes of BCS3. BCS3 was inspected visually using Discovery Studio Visualizer [60]. The study was carried out in a Dell system (3.4 GHz processor, 8 GB RAM, 1 TB hard disk) with Red Hat Linux Enterprise (version 3.0) as an operating system [61,62].

### 3.3. Prediction of In Silico ADMET Properties

The prediction of pharmacokinetic parameters is essential in the drug discovery process to avoid failure during drug development. The prediction of in silico absorption, distribution, metabolism, excretion, and toxicity (ADMET) parameters for the BSC3 was performed by using the Molinspiration Cheminformatics (v2024. 02) server [63]. Further, drug-likeness and ADMET profiling (absorption, solubility, pKa, permeability, bioavailability, volume of distribution, penetration through dermis and eye (ocular), transporter activity, enzymatic interactions, plasma protein binding, half-life, etc.) was predicted with the help of SwissADME (v2.3.0) [64], PreADMET v2.0 [65] and ADMETLab v3.0 [66].

A bioavailability prediction model, called the BOILED-Egg (Brain or IntestinaL EstimateD permeation method) plot was also employed on BCS3, which uses gastrointestinal absorption and blood–brain barrier (BBB) penetration parameters to predict the drug’s bioavailability. Five parameters were used to define the coordinates of ellipses (white and yolk part) of the egg plot: molecular weight, total polar surface area, MLOGP, GI, and BBB. The yolk part represents BBB permeation, whereas the white part of the egg plot represents human intestinal absorption (HIA). A molecule that is plotted outside of the grey region in the graphical plot will be representative of possessing poor absorption and limited BBB penetration. Yolk and white regions are mutually exclusive. The graph also shows if a drug is a P-gp (permeability-glycoprotein) substrate/non-substrate by the appearance of the dot as blue (substrate; PGP+) or red (non-substrate; PGP-).

Similarly, the bioavailability radar provides a graphical representation of the drug-likeness parameters of an orally bioactive drug. The pink area in the graph shows the optimal property range of lipophilicity (LIPO), M.W (SIZE), polarity (POLAR), solubility (INSOLU), saturation (INSATU), and flexibility (FLEX). BCS3 was subjected to the prediction of its bioavailability radar using the SwissADME web server.

The possible metabolites profile after phase I metabolism was also predicted for the compound BCS3, and their possible structures were accessed using BioTransformer v3.0 [67,68]. The metabolic transformation phase was selected as “Phase I (CYP450) transformation” while selecting one number of reaction steps to obtain the predictive metabolic pathways/metabolites for BCS3.

The toxicity profile (in silico) of BCS3 was predicted using ADMETLab, ProTox-II, and Pre-ADMET web servers. These web servers incorporate various parameters like pharmacophores, molecular similarity, fragment propensity, and machine-learning for toxicity prediction endpoints such as organ toxicity, acute toxicity, hERG (human Ether-a-go-go-Related Gene) inhibition, AMES toxicity (mutagenicity), hepatotoxicity, carcinogenicity (rat and mice), cytotoxicity, immunotoxicity, mutagenicity, toxicity targets, adverse outcome pathways, etc. LD_50_ value was predicted using the II web server.

### 3.4. In Vitro Studies

#### 3.4.1. Cell Line, Culture Conditions, and Reagents

The procurement of MDA-MB-231, MCF-7, MDA-MB-468, and MCF-10A cell lines was performed from the National Centre for Cell Science, Pune, India, and the cells were preserved in RPMI-1640 (Gibco, Grand Island, NY, USA) culture medium. The media was supplemented with 10% fetal bovine serum (FBS), 100 µ/mL of penicillin, and 100 mg/mL of streptomycin (37 °C; 5% of CO_2_; 95% of air and humidity) in a humid environment. Different breast cancer cell lines, MCF-7, MDA-MB-231, and MDA-MB-468 were treated with BCS3 according to previously stated methods [69].

#### 3.4.2. In Vitro Cell Cytotoxicity

The cytotoxic potential of BCS3 was quantified through an MTT assay following the instructions of Sahu et al. [70]. BCS3 in its varying concentrations (0.1, 0.2, 0.4, 0.8, 1.6, 3.2, 6.4, 12.8, and 25.6 µM, respectively) was tested against three breast cancer cell lines, MCF-7, MDA-MB-231, and MDA-MB-468, and a normal breast epithelial cell line, MCF-10A for 72 h at 37 °C. The experiments were repeated thrice with every concentration.

#### 3.4.3. Western Blot Analysis for the Determination of Protein Expression of Apoptotic Proteins in MDA-MB-231 Cells

To examine the in vitro expressions of critical apoptotic proteins (XIAP, cIAP1, cIAP2, p53, MCL-1, Apaf-1, caspase-8, caspase-3, and caspase-9) in MDA-MB-231 cells post-BCS3 (0.8, 1.6, and 3.2 µM, respectively) treatment, the western blotting technique was implemented as per the instructions followed by Sahu et al. [69]. The respective proteins were initially separated using SDS-PAGE followed by electrophoretic transfer into nitrocellulose membranes (Millipore) from gels. The membranes were blocked for 2 h followed by consecutive incubation with primary and secondary antibodies for the detection of specific proteins. The protein bands were then observed using an enhanced chemiluminescence solution (San Francisco, CA, USA) and a densitometric analysis was performed with the aid of Image J software v1.54k (NIH, Washington, WA, USA). β-actin, α-tubulin, and p-97 were considered as internal control.

#### 3.4.4. Immunofluorescence Analysis of Bax, Bcl-2, Caspase-8, and Caspase-3 in MDA-MB-231 Cells

The estimation of in vitro expression of apoptotic proteins (Bax, Bcl-2, caspase-8, and 3) in MDA-MB-231 cells was observed with the immunofluorescence technique as per the protocol followed by Sahu et al. [71]. After treatment with 1.6 and 3.2 µM of BCS3, tumor cells were fixed with 4% paraformaldehyde. Then, the cells were blocked by treatment with donkey serum in PBST (Sigma Aldrich, St. Louis, MO, USA) for 1 h. Following blockage, Bax primary antibody (Santa Cruz Biotech, Dallas, TX, USA), Bcl-2 primary antibody (Abcam, Cambridge, UK), caspase-3 primary antibody (Abcam, Cambridge, UK), or caspase-8 primary antibody (Abcam, Cambridge, UK) was added to the cells (1:200) in PBST overnight (4 °C) followed by further treatment with conjugated secondary antibodies (FITC, Alexa 647 and Alexa 633, Millipore, Watford, UK) (1:400). Lastly, the cells were incubated with Hoechst and DAPI stains for 10 min for nuclei staining. The sample was then examined with the aid of FLOWVIEW (Waltham, MA, USA), an Olympus confocal microscope.

#### 3.4.5. Estimation of Intracellular ROS Generation

The changes in the levels of intracellular ROS production were evaluated using a dichlorodihydro-fluorescein diacetate (DCFH-DA) analysis kit (Abcam) as per the manufacturer’s instructions. MDA-MB-231 cells were seeded primarily in 96-well plates followed by successive treatment with 0.8, 1.6, and 3.2 µM of BCS3, respectively. The media was removed, followed by the incubation of cells with 10 µM DCFH-DA and 100 µL of Hanks’ buffered salt (HBBS) solution at 37 °C for 30 min. ROS levels were measured using a spectrofluorometer [72].

#### 3.4.6. Determination of ATP Content Determination

Following the protocol given by Shafiei et al. [73], the cellular ATP content was determined. MDA-MB-231 cell seeding was performed at a density of 5 × 10^5^ cells/well in 6-well plates for 24 h, followed by treatment with BCS3 (0.8, 1.6, and 3.2 µM concentrations). Fresh medium was taken as a control and the media were incubated for the next 24 h. After trypsinization, the cells were washed twice using cold phosphate-buffered saline (PBS) and then were tested for the determination of ATP as per the manufacturer’s instructions using an ATP assay kit (Abcam ab83355).

#### 3.4.7. Determination of LDH Release

The release of lactate dehydrogenase (LDH) was evaluated in the medium that enzymatically illustrates cell injury, necrosis/apoptosis, or membrane integrity of cells. To access the cytotoxic potential of BCS3 through the quantification of LDH release, the CytoTox 96 Non-Radioactive Cytotoxicity Assay Kit (Promega, Madison, WI, USA, G1780) was utilized. The LDH release from MDA-MB-231 cells was calculated post-treatment with BCS3 (0.8, 1.6, and 3.2 µM). Equal proportions of culture medium and LDH reagent (50 µL) were mixed and incubated at room temperature for 30 min. The absorbance value was examined at 490 nm [74].

#### 3.4.8. Evaluation of Mitochondrial Membrane Potential

The MDA-MB-231 cells were incubated with JC-1 and JC-10 dye-loading solutions (Abcam, Cambridge, MA, USA along with varying concentrations of BCS3 (0.8, 1.6, and 3.2 µM) for 30 min. The formation of monomeric forms and J-aggregates of JC-1 and JC-10 was evaluated by measuring the fluorescent intensities of the media. The calculations were obtained using a microplate reader at Ex/Em = 490/525 nm and 490/590 nm [75].

#### 3.4.9. Annexin V Staining and Flow Cytometry for the Analysis of Apoptosis

MDA-MB-231 cells are treated with predetermined concentrations of BCS3 with a cell density of 2 × 10^6^ cells for 24 h. The cells were then treated with trypsin, washed with PBS, and resuspended using 100 µL of annexin binding buffer (Thermo Fisher Scientific, Waltham, MA, USA, V13246) consisting of 1 mM of CaCl_2_, 140 mM of NaCl, 0.75 mM of MgCl_2_, 4 mM of KCl, and 10 mM of HEPES (4-(2-hydroxyethyl)piperazine-1-ethanesulfonic acid) in double-distilled water (DDW). The suspended cells were then stained with annexin V-Alexa Fluor 647 (BD Biosciences, San Diego, CA, USA) for the next 30 min at 4 °C. The cells were then thoroughly washed and treated with propidium iodide (PI). The cells were then instantly analyzed for the evaluation of results using the CytoFLEX flow cytometer (Beckman Coulter, Brea, CA, USA) and CytoExpert Software v2.6 (Beckman Coulter Life Sciences) [76].

#### 3.4.10. Cell Cycle Analysis

MDA-MB-231 cells were treated with BCS3 (0.8, 1.6, and 3.2 µM) for 72 h followed by harvesting and fixing the cells at 4 °C with 75% ethanol overnight. The fixed cells were then collected and suspended using PBS buffer with propidium bromide (PI) (10 µg/mL) and RNAase (10 µg/mL). The suspension was then incubated at room temperature for 30 min. The CytoFLEX flow cytometer operated with CytoExpert Software was used to measure total gated cell percentage and construct a histogram [77].

#### 3.4.11. Analysis of Apoptotic and Cell Cycle Proteins Through ELISA

The protein expressions of MDM2, cytochrome c, p21, phospho-CDK1 and phospho-cyclin B1 on MDA-MB-231 cells after BCS3 treatment were quantified through ELISA by following the instructions provided by manufacturers of the respective colorimetric kits (cytochrome c (SunLong Biotech Co., Hangzhou, China), MDM2 ELISA kit, MyBioSource (MBS2022201), abcam Human p21 ELISA kit, (ab214658), abcam Phospho-CDK1 (T161) ELISA kit (ab279756), and abcam Phospho-cyclin B1 (S126) ELISA kit (ab279766)) and the microplate spectrophotometer (Thermo Scientific, MULTISKAN GO, MA, USA).

#### 3.4.12. Quantitative Real-Time PCR (qPCR) Analysis of Critical Apoptotic Proteins

The extraction of total RNA from MDA-MB-231 cell lines was performed using TRI reagent (Sigma-Aldrich), and then it was purified using a mixture of DDW (150 µL), glycogen (3 µL), sodium acetate (20 µL; 3 mol/L), and absolute ethanol (600 µL). The RNA mixture was then stored for 2 h at −80 °C and then centrifuged for 15 min at 13,250× *g* (4 °C) to acquire purified RNA required for the synthesis of cDNA. A cDNA synthesis kit (Bio-Rad, Hercules, CA, USA) was used for synthesizing the first stranded cDNA by adding RNA (1 µg) and oligo dTTP following manufacturer guidelines. The amplification of equivalent portions of cDNA was performed in a 20 µL reaction employing 2x SYBR qPCR Matrix Mix (Kapa-Biosystems, Wilmington, MA, USA) and specific primers. The qPCR of all genes was performed by employing their respective sense and anti-sense primers for the following as mentioned in Table 3. The cycles for the given genes were performed as 1 cycle (180 s; 95 °C), 39 cycles in each 95 °C (10 s) and 58 °C (30 s). The presence of a single peak as an outcome from melting curve analyses (60.0–95.0 °C; increment 0.5 °C, for 0.05 s) proved to be a verification of validation of PCR amplification. β-actin was chosen as internal control, and each of the experiments was reproduced thrice.

### 3.5. In Vivo Studies

#### 3.5.1. DMBA-Induced Breast Cancer Model in Rats

Virgin nulliparous Sprague-Dawley (SD) rats (50–55 days old) were procured from the National Institute of Nutrition (NIN), Hyderabad, and were maintained at the Central Animal Facility of Birla Institute of Technology, Mesra, Ranchi, India (1968/PO/Re/17/CPCSEA). This study was approved, having protocol approval number 1972/PH/BIT/105/20/IAEC by the Institutional Animal Ethics Committee (IAEC). Animal maintenance was performed as per standard guidelines, and the animals were provided with the standard pelleted diet and water ad libitum. Prior to the conducting of experiments through ethical standards, the animals were acclimatized to the laboratory conditions in polystyrene cages for a week. Any possible toxicity of BCS3 was evaluated by the administration of single BCS3 doses in increasing order in SD rats as per OECD guidelines [78]. The animals were monitored for a consecutive 14 days for any visible toxic effects or morbidity. Treatment with a single BCS3 dose did not elicit any toxicity or morbidity up to a 300 mg/kg dose. Therefore, 15 and 30 mg/kg b.w doses were selected for evaluation of antitumor activity.

The evaluation of the antitumor potential of BCS3 was performed following the methods of Bose et al. [76]. Twenty-four SD rats were obtained from the animal house facility and randomly distributed into four groups (n = 6/group): group I (control/SD/DMBA −/−), group II (SD/DMBA +/+), group III (SD/DMBA +/+ BCS3 15 mg/kg), and group IV (SD/DMBA +/+ BCS3 30 mg/kg). The initiation and development of breast carcinogenesis was established by single subcutaneous dose administration of 20 mg 7,12-dimethylbenz(a)anthracene (DMBA) suspended in 0.5 mL of olive oil into the air pouch produced by the air-pouch technique. The tumor location was monitored by palpation on a weekly basis. After 90 days of cancer induction, animals were orally administered with 15 and 30 mg/kg b.w (groups III and IV) of BCS3 for the consecutive 28 days. Changes in body weight were recorded weekly throughout the experimental period. After the completion of 118 days of the study protocol, the animals were sacrificed by cervical dislocation under anesthesia followed by the isolation of mammary tissues and tumor tissues. The tumor size was measured using a caliper, and the tumor volume was calculated (ellipsoid shape of tumor) using the following formula: V = 4/3πr12.r2 (r1: short radius; r2: long radius) [70]. The tissues were sliced into thin sections (2–4 mm) and stored in liquid N_2_ for immunofluorescence microscopy.

#### 3.5.2. Histopathological Analysis of Mammary/Tumor Tissues

After animal euthanasia, the breast and tumor tissues were isolated and preserved in 10% buffered formalin. The tissues were then infixed in paraffin wax and cut into fine sections (6–8 µm) using a rotary microtome. Following standard protocols, the tissue sections were stained with hematoxylin and eosin dyes [79].

#### 3.5.3. In Vivo Quantification of Apoptosis Proteins by ELISA

The levels of cell cycle proteins, p21, phospho-CDK1, phospho-cyclin B1, and XIAP (DuoSet IC: Cat. No. DYC822 R&D Systems) were evaluated in tissue homogenates obtained from the excised mammary and tumor tissues of the experimental animals using the above-mentioned ELISA kits of respective proteins as per manufacturer’s instructions.

#### 3.5.4. In Vivo Apoptosis Study Through TUNEL Assay

TUNEL assay was performed on the 30 mm breast tumor tissues embedded in paraffin collected from experimental animals for the determination of intratumoral-late-stage apoptosis. This was followed by tissue staining with ClickiT plus the TUNEL assay kit (Thermo-Fischer, Gibco, MA, USA) that included Alexa Fluor 594 as an in-situ detector for apoptosis. The manufacturer’s instructions were followed, and the tissues were examined under a confocal microscope (FLOWVIEW, Olympus).

#### 3.5.5. Immunofluorescence Analysis of Cleaved Caspase-3 and Bax Proteins in Mammary Tissues

To examine the effect of BCS3 on the expression of cleaved caspase-3 and Bax proteins, an immunofluorescence analysis was carried out on mammary tumors excised from cancer-bearing animals. A total of 2–3 mm of finely sliced mammary tissues were treated with ornithine decarboxylase media and kept in liquid N_2_ for a while. The tissues were cryo-sectioned in 5 µm sections and stored in cold buffered formalin (4% *v*/*v*) for 10 min, followed by a minute’s treatment with 1X OPTIMAX. The mammary tissue sections were then stained with respective primary antibodies (1 mg/mL) of cleaved caspase-3 and Bax (Santa Cruz Biotech, USA) incubated at 25 °C for 1 h. Further, the slides were rinsed twice for one minute each with 1X OPTIMAX and incubated again in the dark for 30 min with corresponding antibodies (Bangalore, Genei, India) and with added ice. Again, the slides were rinsed twice for one minute each using 1X OPTIMAX before coverslips were placed over the slides. Lastly, the slides were examined under the FLOWVIEW, Olympus confocal microscope.

### 3.6. Combinatorial Synergy Study of BCS3 with Doxorubicin in MDA-MB-231 Cells

For the quantification of drug combinatory effects between BCS3 and doxorubicin, a synergy assessment was performed using Synergyfinder 3.0 [80] utilizing the zero interaction potency (ZIP) reference model (synergy score: <−10: antagonism; −10 to 10: additivity; >10: synergism) [81]. MDA-MB-231 cells were treated with BCS3 doses of 0, 0.25, 0.5, 1, 3, 5, 10, and 15 µM, and doxorubicin doses of 0, 0.0325, 0.065, 0.125, 0.25, 0.5, 1, and 3 µM for 24 h. After adding MTS to the wells for 2 h, the absorbance value was recorded at 495 nm with a microplate reader.

### 3.7. In Vivo Sub-Acute Toxicity Analysis Following OECD 407 Guidelines

For the evaluation of the potential side effects of BCS3, a repeated-dose toxicity study was conducted as per the mentioned protocol of OECD guidelines 407 [82]. Wistar rats were used for the study (n = 10/group). BCS3 was dissolved in absolute DMSO (1% *w*/*v*) made up to the desired concentration with distilled water and administered orally daily (volume: 1 mL/100 g of rat b.w) at 30 and 300 mg/kg doses as per the study planned by Beladiya et al. [83]. Twenty-five animals were obtained for the study (n = 5/group), where animals were randomly assorted into the following groups: Group I (control, 10% DMSO), groups II and III (30 and 300 mg/kg oral doses of BCS3, respectively, for 28 days) and satellite groups, groups IV (control) and V (300 mg/kg BCS3 for 28 days) followed by an additional 14 days (total 42 days) of no drug administration for follow-up observations. The satellite groups were designed for the determination of recovery or reversibility from toxic effects caused by the administration of test substances.

Initial body weights were recorded on a weekly basis. The satellite group animals were observed for an additional 14 days for persistence, reversibility, or delayed occurrence of any adverse drug effects post-sub-acute treatment. The experimental animals were continuously observed for monitoring toxicity signs, such as ruffled hair, anorexia, weight loss, skin ulceration, diarrhea, and toxic death. At the end of the experimental study protocol (29th day for groups I, II, and III, and 43rd day for the satellite group), animals were anesthetized and blood was collected by cardiac puncture using a disposable syringe. The blood was collected in K2EDTA tubes for the analyses of hematological parameters: hemoglobin, red blood corpuscles (RBC), white blood corpuscles (WBC), platelets count, lymphocytes, neutrophils, monocytes, eosinophils, and basophils. For the analysis of biochemical parameters, blood was collected and stored in plain tubes (non-heparinized) at room temperature for 15 to 20 min. It was then allowed to coagulate, followed by centrifugation at 5000 rpm at 4 °C for 15 min to extract the serum for analysis. Biochemical parameters like liver function markers, aspartate aminotransferase (AST), alanine aminotransferase (ALT), alkaline phosphatase (ALP), lactate dehydrogenase (LDH), and gamma-glutamyl transferase (γ-GT) were analyzed for toxicity in experimental animals.

Immediately after blood withdrawal, the animals were humanely euthanized by cervical dislocation to isolate the vital organs (liver, kidney, brain, and lungs) for macroscopical and histological examination. Organ tissue sections were prepared [79] and examined under the Leica microscope (Leica DME microscope, Wetzlar, Germany).

### 3.8. Statistical Analysis

The data sets were statistically compared by one-way ANOVA using Bonferroni’s multiple comparison test having similar sample size numbers. The data sets were represented using mean ± standard error mean (SEM) and the level of significance was set at *p* < 0.05.

## 4. Conclusions

In a concluding remark, the anti-proliferative and anti-tumor potential of synthesized azomethine scaffold, BCS3, was elucidated against in vitro and in vivo breast cancer cells by antagonizing IAPs to regulate MDM2-p53 and Bcl-2-caspase axes that potentiated intrinsic and extrinsic apoptotic pathways after the determination of its promising binding affinity with the BIR3 domain of IAPs through in silico molecular docking studies. IAP inhibition also caused p53-mediated p21-CDK1-cyclin B1 inhibition causing cell cycle arrest at S and G2/M phases. The therapeutic dose of BCS3 (30 mg/kg) was rendered safe and highly efficient after in vivo toxicological evaluation. Remarkable synergy was obtained between the drug combinations of BCS3 with doxorubicin. Therefore, the study revealed the cytotoxic and anti-proliferative effects of BCS3 as a highly efficacious and less toxic candidate for breast cancer therapy.

## Figures and Tables

**Table 1:** Binding interactions of **BCS3** and the reference co-crystal ligands with the inhibitors of apoptosis proteins (IAPs).

Inhibitors of Apoptosis Proteins (IAPs)	Binding Affinity ΔG (Kcal mol^−1^) of Compounds	Amino Acid Residues Interacting with BCS3	
		H-Bonding (Distance in Å)	Hydrophobic Interaction (π—π Stacking)
**XIAP (3CLX)**	BCS3: −7.8 Co-crystal ligand (X22): −8.3	C-Tyr 324 (3.52438 Å)	Trp323, Tyr324,
**cIAP1 (3MUP)**	BCS3: −6.9 Co-crystal ligand (SMK): −7.9	C-Cys309 (2.60077 Å)	Phe324, Trp323
**cIAP2 (3M0A)**	BCS3: −5.7	H-Glu292 (2.8456 Å)	Leu289, Val293, Val296

XIAP: X-linked inhibitor of apoptosis protein; cIAP: Cellular inhibitor of apoptosis protein; X22: (3S,6S,7S,9aS)-6-{[(2S)-2-aminobutanoyl]amino}-*N*-(diphenylmethyl)-7 (hydroxymethyl)-5-oxooctahydro-1*H*-pyrrolo [1,2-*a*]azepine-3-carboxamide; SMK: (3S,6S,7R,9aS)-6-{[(2S)-2-aminobutanoyl]amino}-7-(2-aminoethyl)-*N*-(diphenylmethyl)-5-oxooctahydro-1*H*-pyrrolo[1,2-*a*]azepine-3-carboxamide.

**Table 2:** In silico predicted ADMET properties of BCS3.

ADME Properties (In Silico)			Toxicity Profile (In Silico)		
**(a) Physicochemical Properties: bioavailability**			**i. Organ Toxicity**		
Pure water solubility (mg/mL)	6.989		Hepatotoxicity (H-HT)	0.056	>0.5: Toxic; <0.5: Non-toxic
Buffer solubility (mg/mL)	1.7944				
**(b) Absorption**			**ii. Toxicological endpoints**		
CaCo-2 permeability (CCP, nm/s)	56.4911	+: Good permeability	Carcinogenicity	0.216	>0.5: Carcinogenic; <0.5: Non-carcinogenic
Human intestinal absorption (HIA, %)	100	70–100: Intestinally well absorbed	Cytotoxicity	-	
MDCK (nm/s)	1.926	<25: Poor permeability; >500: Great permeability	Immunotoxicity	-	
Skin permeability (cm/h)	−2.261	+: Permeable through dermis; −: Impermeable through dermis	Eye corrosion	0.015	>0.5: Corrosive; <0.5: Non-corrosive
			Respiratory toxicity	0.075	>0.5: Toxic; <0.5: Non-toxic
**(c) Distribution**			**iii. Toxicological pathways**		
PPB	100%	>90%: Highly protein bound	Aryl hydrocarbon receptor (AhR)	0.443 (+)	>0.5: Positive; <0.5: Negative
BBB	0.974	>0.1: BBB permeable; <0.1: BBB impermeable	Androgen receptor (AR)	0.015	>0.5: Positive; <0.5: Negative
Volume of distribution	0.456	>0.7 L/kg: Lipophilic and bound to tissue; <0.7: Hydrophilic and bound to plasma	Androgen receptor–ligand-binding domain (AR-LBD)	0.005	>0.5: Positive; <0.5: Negative
			Estrogen receptor alpha (ERα)	0.163	>0.5: Positive; <0.5: Negative
			Estrogen receptor–ligand-binding domain	0.211	>0.5: Positive; <0.5: Negative
			Peroxisome proliferator-activated receptor gamma (PPAR-γ)	0.004	>0.5: Positive; <0.5: Negative
**(d) Metabolism**			**iv. Stress-response pathways**		
CYP450 1A2 substrate	0.318	>0.5: Substrate; <0.5: Non-substrate	Nuclear factor (erythroid derived 2)-like 2/antioxidant responsive element (nrf2/ARE)	0.816	>0.5: Positive; <0.5: Negative
CYP450 1A2 inhibitor	0.394	>0.5: Inhibitor; <0.5: Non-inhibitor	Heat shock protein element	0.033	>0.5: Positive; <0.5: Negative
CYP450 3A4 substrate	0.206	>0.5: Substrate; <0.5: Non-substrate	Mitochondrial membrane potential (MMP)	0.834	>0.5: Positive; <0.5: Negative
CYP450 3A4 inhibitor	0.104	>0.5: Inhibitor; <0.5: Non-inhibitor	ATPase family AAA-domain containing protein (ATAD5)	0.394	>0.5: Positive; <0.5: Negative
CYP450 2C9 substrate	0.149	>0.5: Substrate; <0.5: Non-substrate			
CYP450 2C9 inhibitor	0.296	>0.5: Inhibitor; <0.5: Non-inhibitor			
CYP450 2C19 substrate	0.064	>0.5: Substrate; <0.5: Non-substrate			
CYP450 2C19 inhibitor	0.437	>0.5: Inhibitor; <0.5: Non-inhibitor			
CYP450 2D6 substrate	0.385	>0.5: Substrate; <0.5: Non-substrate			
CYP450 2D6 inhibitor	0.223	>0.5: Inhibitor; <0.5: Non-inhibitor			
**(e) Excretion**			**v. Predictive toxicity**		
T_1/2_	1.697	>8H: High; 3–8H: Moderate; <3H: Low	hERG inhibition	0.261	>0.5: Blocker; <0.5: Non-blocker
Clearance	1.572	>15: High; 5–15: Moderate; <5: Low	Skin sensitization	0.192	>0.5: Positive; <0.5: Negative

CCP: Caco-2 cell permeability; MDCK: Madin-Darby canine kidney; PPB: plasma protein binding; BBB: blood–brain barrier; hERG: human ether-a-go-go-related gene.

**Table 3:** Forward and backward primer sequences of messenger RNA for real-time quantitative polymerase chain reaction (RT-qPCR) analysis.

	mRNA	Primer	Sequence (5′ > 3′)
1	XIAP	F	TCCAGAATGGTCAGTACAAAGTTG
		R	TTTGTTGAATTTGGGAAATTCCT
2	cIAP1	F	AGGTGTGAGTTCTTGATACGAA
		R	TTGTTTCACCAGGTCTCTATTA
3	cIAP2	F	AGGTGTTGGGAATCTGGAGAT
		R	GCAGCATTAATCACAGGAGTA
4	MDM2	F	GGCGTGCCAAGCTTCTCTGTG
		R	ACCTGAGTCCGATGATTCCTGCTG
5	Survivin	F	GCCATGAATTCATGGGTGCCCCGACGTTGC
		R	AGCTCTCTAGAGAGGCCTCAATCCATGGCA
6	p53	F	GCGCACAGAGGAAGAGAATC
		R	CTCTCGGAACATCTCGAAGC
7	Bax	F	TGCCAGCAAACTGGTGCTCA
		R	GCACTCCCGCCACAAAGATG
8	Bak	F	CGTTTTTTACCGCCATCAGCAG
		R	ATAGCGTCGGTTGATGTCGTCC
9	Bcl-2	F	CGCATCAGGAAGGCTAGAGT
		R	AGCTTCCAGACATTCGGAGA
10	Bcl-xL	F	CCCTTCAGAATCTTATCTTGGCT
		R	GGGAAAGCTTGTAGGAGAGAAAG
11	MCL-1	F	CCAAGAAAGCTGCATCGAACCAT
		R	CAGCACATTCCTGATGCCACCT
12	Caspase-8	F	CATCCAGTCACTTTGCCAGA
		R	GCATCTGTTTCCCCATGTTT
13	Caspase-3	F	GAACTGGACTGTGGCATTGA
		R	TGTCGGCATACTGTTTCAGC
14	Caspase-9	F	TGGACGACATCTTTGAGCAG
		R	GCAAGATAAGGCAGGGTGAG
15	p21	F	CTTTGTCACCGAGACACCAC
		R	CAGGTCCACATGGTCTTCCT
16	CDK1	F	TATGTATCGAACTGCGA
		R	CTCTATGGCGCTAACT
17	Cyclin B1	F	TTATGACCCTGGATCTGTA
		R	CTCTCATCGCCATTCAGTGAA
18	β-actin	F	AGCCATGTACGTAGCCATCC
		R	TCCCTCTCAGCTGTGGTGGTGAA

CDK: Cyclin-dependent kinase; Bcl-2: B-cell lymphoma-2; Bcl-xL: B-cell lymphoma-extra-large; XIAP: X-linked inhibitor of apoptosis protein; cIAP: cellular inhibitor of apoptosis protein; F: forward; R: reverse.

## Data Availability

The data supporting this article have been included as part of the Supplementary Information.

## Supplementary Materials

The following supporting information can be downloaded at: https://www.mdpi.com/article/10.3390/ph17121645/s1, Document S1 (.ppt): Supporting information of experimental details. Experimental details of synthesis (by condensation reaction) and characterization of BCS3 by mass spectrometry, NMR (1HNMR and 13CNMR), IR spectrometry, and HPLC trace. In silico analysis (molecular docking, metabolic profile of BCS3), in vitro mRNA expression of critical proteins, and in vivo toxicity profile (body weight, hematological and biochemical parameters) are included in Figures S1–S7 and Tables S1 and S2. Document S2 (.excel): Full blots of western blotting analysis of proteins used in in vitro analysis. Full western blots of the following proteins, XIAP, cIAP1, cIAP2, p53, MCL-1, SMAC, Survivin, Apaf-1, caspase-8, caspase-3, and caspase-9.

## Abbreviations

BBB: blood–brain barrier; CDK: cyclin-dependent kinase; cIAP1/2: cellular inhibitor of apoptosis 1 and 2; DILI: drug-induced liver injury; DMBA: 7,12-dimethylbenz(a)anthracene; FACS: fluorescence activating cell sorting; HIA: human intestinal absorption; IAPs: inhibitors of apoptosis; LDH: lactate dehydrogenase; MCL-1: myeloid cell leukemia sequence-1; MMP: mitochondrial membrane potential; PI: propidium iodide; SMAC: secondary mitochondria-derived activator of caspases; ROS: reactive oxygen species; TUNEL: terminal deoxynucleotidyl transferase biotin dUTP nick end labeling; XIAP: X-linked inhibitor of apoptosis.
