# Supplementary material for: An Azomethine Derivative, BCS3, Targets XIAP and cIAP1/2 to Arrest Breast Cancer Progression Through MDM2-p53 and Bcl-2-Caspase Signaling Modulation
Source: Pharmaceuticals (Basel). 2024 Dec 6;17(12):1645. doi: 10.3390/ph17121645 (PMC11678930; doi:10.3390/ph17121645)
Supplement: Supplementary file 1 [file pharmaceuticals-17-01645-s001.zip › ESI 1.pptx]

## Slide 1
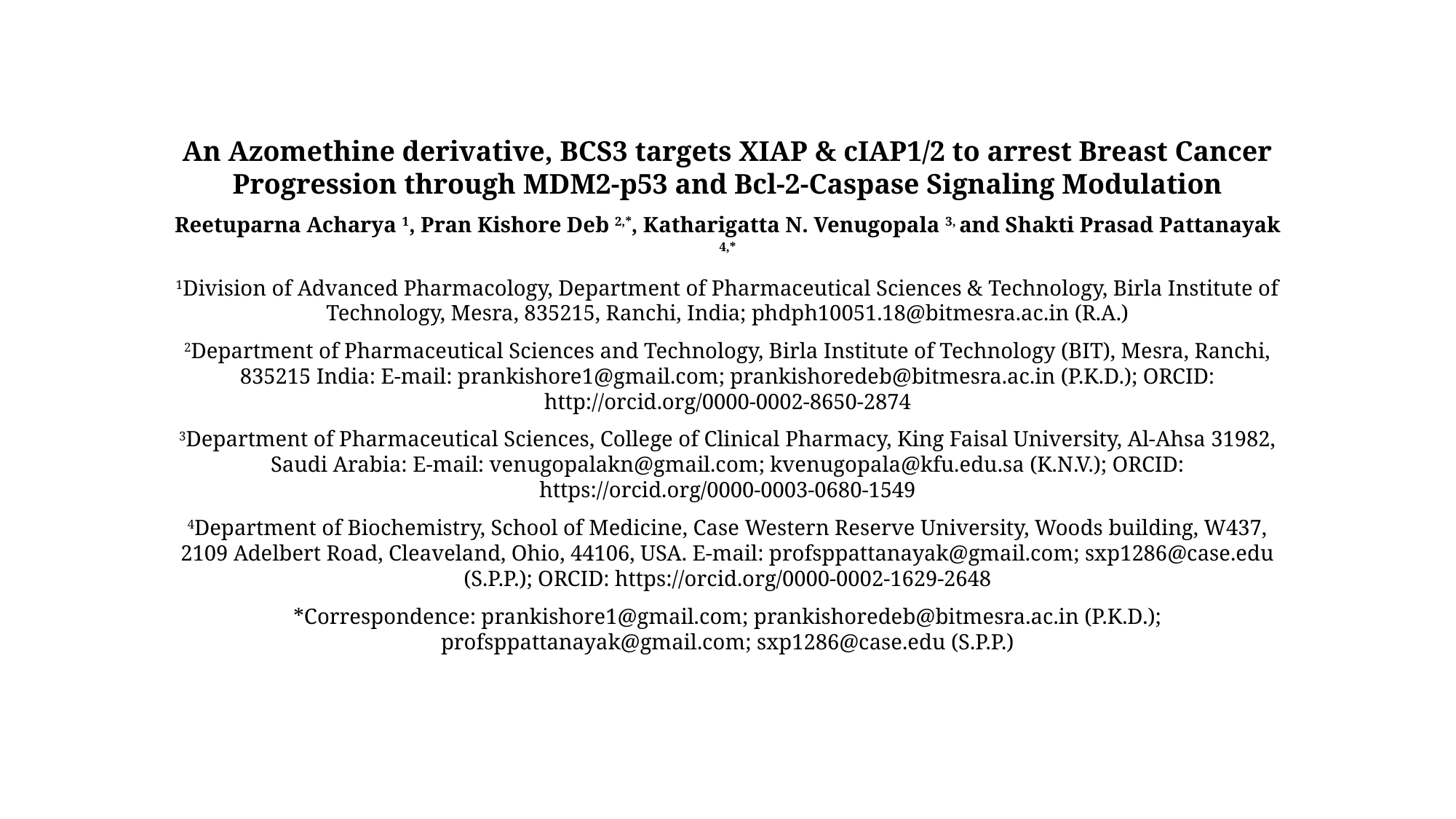

An Azomethine derivative, BCS3 targets XIAP & cIAP1/2 to arrest Breast Cancer Progression through MDM2-p53 and Bcl-2-Caspase Signaling Modulation
Reetuparna Acharya 1, Pran Kishore Deb 2,*, Katharigatta N. Venugopala 3, and Shakti Prasad Pattanayak 4,*
1Division of Advanced Pharmacology, Department of Pharmaceutical Sciences & Technology, Birla Institute of Technology, Mesra, 835215, Ranchi, India; phdph10051.18@bitmesra.ac.in (R.A.)
2Department of Pharmaceutical Sciences and Technology, Birla Institute of Technology (BIT), Mesra, Ranchi, 835215 India: E-mail: prankishore1@gmail.com; prankishoredeb@bitmesra.ac.in (P.K.D.); ORCID: http://orcid.org/0000-0002-8650-2874
3Department of Pharmaceutical Sciences, College of Clinical Pharmacy, King Faisal University, Al-Ahsa 31982, Saudi Arabia: E-mail: venugopalakn@gmail.com; kvenugopala@kfu.edu.sa (K.N.V.); ORCID: https://orcid.org/0000-0003-0680-1549
4Department of Biochemistry, School of Medicine, Case Western Reserve University, Woods building, W437, 2109 Adelbert Road, Cleaveland, Ohio, 44106, USA. E-mail: profsppattanayak@gmail.com; sxp1286@case.edu (S.P.P.); ORCID: https://orcid.org/0000-0002-1629-2648
*Correspondence: prankishore1@gmail.com; prankishoredeb@bitmesra.ac.in (P.K.D.); profsppattanayak@gmail.com; sxp1286@case.edu (S.P.P.)

## Slide 2
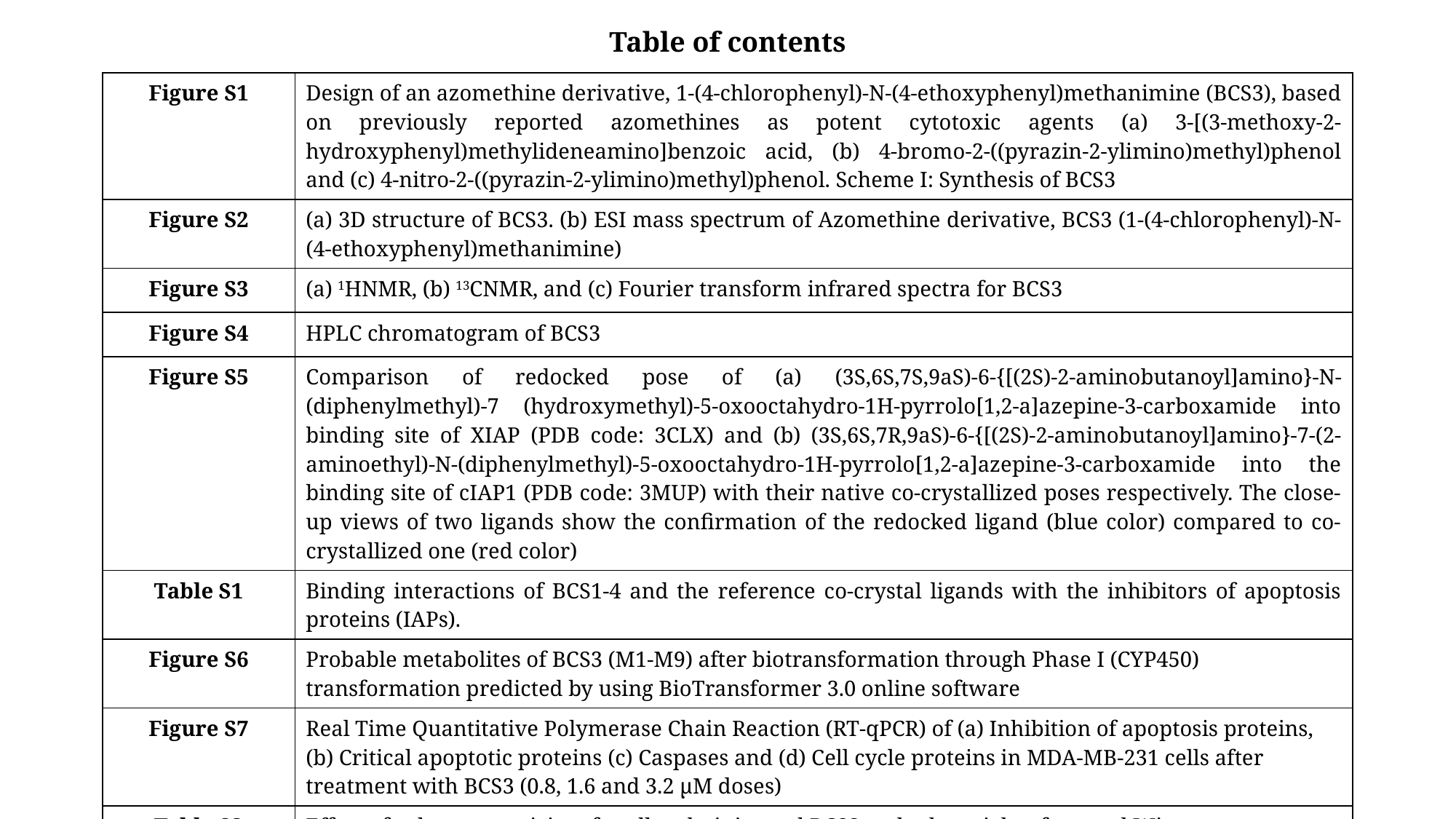

Table of contents
| Figure S1 | Design of an azomethine derivative, 1-(4-chlorophenyl)-N-(4-ethoxyphenyl)methanimine (BCS3), based on previously reported azomethines as potent cytotoxic agents (a) 3-[(3-methoxy-2-hydroxyphenyl)methylideneamino]benzoic acid, (b) 4-bromo-2-((pyrazin-2-ylimino)methyl)phenol and (c) 4-nitro-2-((pyrazin-2-ylimino)methyl)phenol. Scheme I: Synthesis of BCS3 |
| --- | --- |
| Figure S2 | (a) 3D structure of BCS3. (b) ESI mass spectrum of Azomethine derivative, BCS3 (1-(4-chlorophenyl)-N-(4-ethoxyphenyl)methanimine) |
| Figure S3 | (a) 1HNMR, (b) 13CNMR, and (c) Fourier transform infrared spectra for BCS3 |
| Figure S4 | HPLC chromatogram of BCS3 |
| Figure S5 | Comparison of redocked pose of (a) (3S,6S,7S,9aS)-6-{[(2S)-2-aminobutanoyl]amino}-N-(diphenylmethyl)-7 (hydroxymethyl)-5-oxooctahydro-1H-pyrrolo[1,2-a]azepine-3-carboxamide into binding site of XIAP (PDB code: 3CLX) and (b) (3S,6S,7R,9aS)-6-{[(2S)-2-aminobutanoyl]amino}-7-(2-aminoethyl)-N-(diphenylmethyl)-5-oxooctahydro-1H-pyrrolo[1,2-a]azepine-3-carboxamide into the binding site of cIAP1 (PDB code: 3MUP) with their native co-crystallized poses respectively. The close-up views of two ligands show the confirmation of the redocked ligand (blue color) compared to co-crystallized one (red color) |
| Table S1 | Binding interactions of BCS1-4 and the reference co-crystal ligands with the inhibitors of apoptosis proteins (IAPs). |
| Figure S6 | Probable metabolites of BCS3 (M1-M9) after biotransformation through Phase I (CYP450) transformation predicted by using BioTransformer 3.0 online software |
| Figure S7 | Real Time Quantitative Polymerase Chain Reaction (RT-qPCR) of (a) Inhibition of apoptosis proteins, (b) Critical apoptotic proteins (c) Caspases and (d) Cell cycle proteins in MDA-MB-231 cells after treatment with BCS3 (0.8, 1.6 and 3.2 µM doses) |
| Table S2 | Effect of sub-acute toxicity of orally administered BCS3 on body weight of normal Wistar rats |
| Table S3 | Sub-acute toxicity of orally administered BCS3 on haematological and biochemical parameters of Wistar rats |

## Slide 3
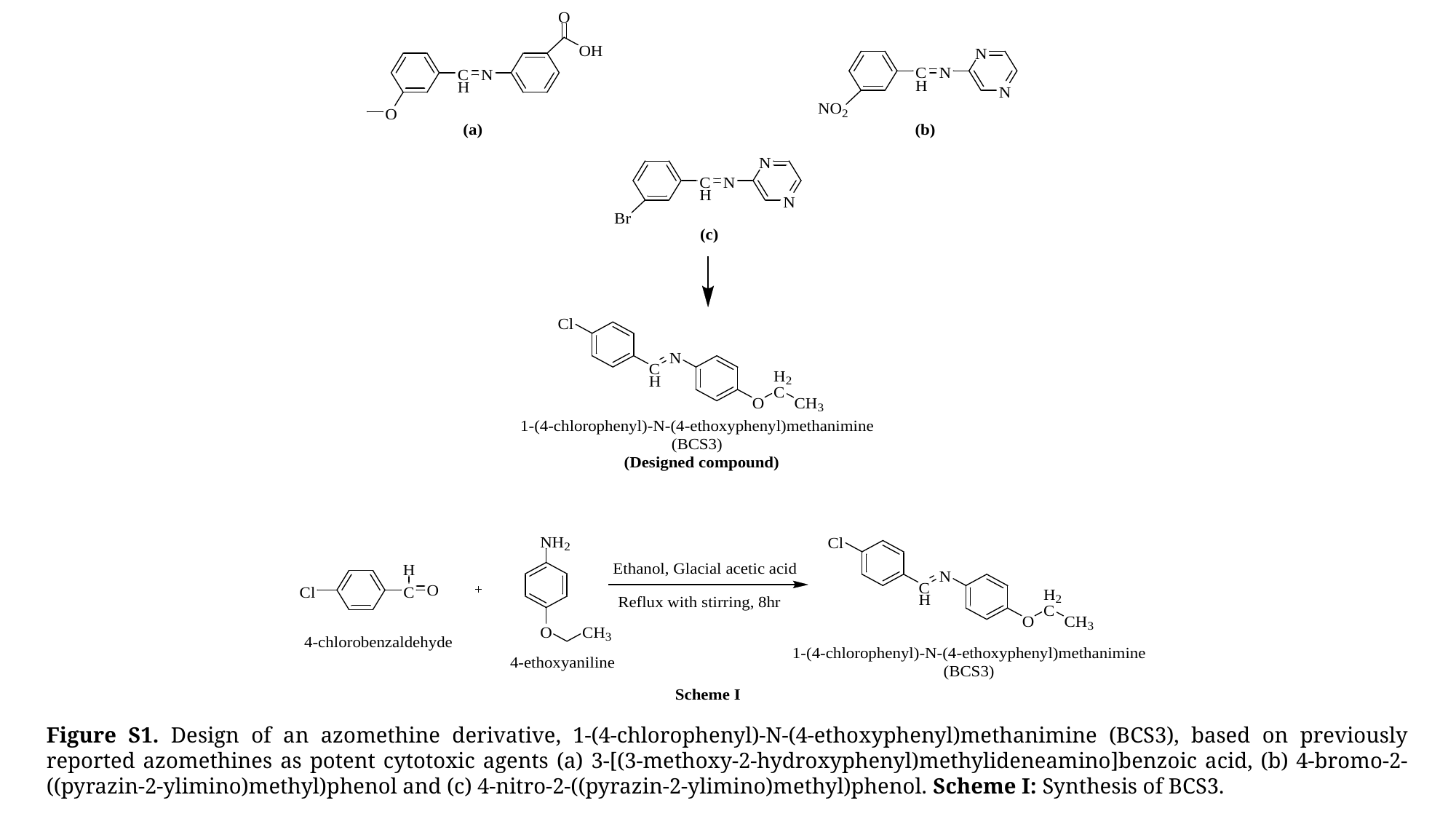

Figure S1. Design of an azomethine derivative, 1-(4-chlorophenyl)-N-(4-ethoxyphenyl)methanimine (BCS3), based on previously reported azomethines as potent cytotoxic agents (a) 3-[(3-methoxy-2-hydroxyphenyl)methylideneamino]benzoic acid, (b) 4-bromo-2-((pyrazin-2-ylimino)methyl)phenol and (c) 4-nitro-2-((pyrazin-2-ylimino)methyl)phenol. Scheme I: Synthesis of BCS3.

## Slide 4
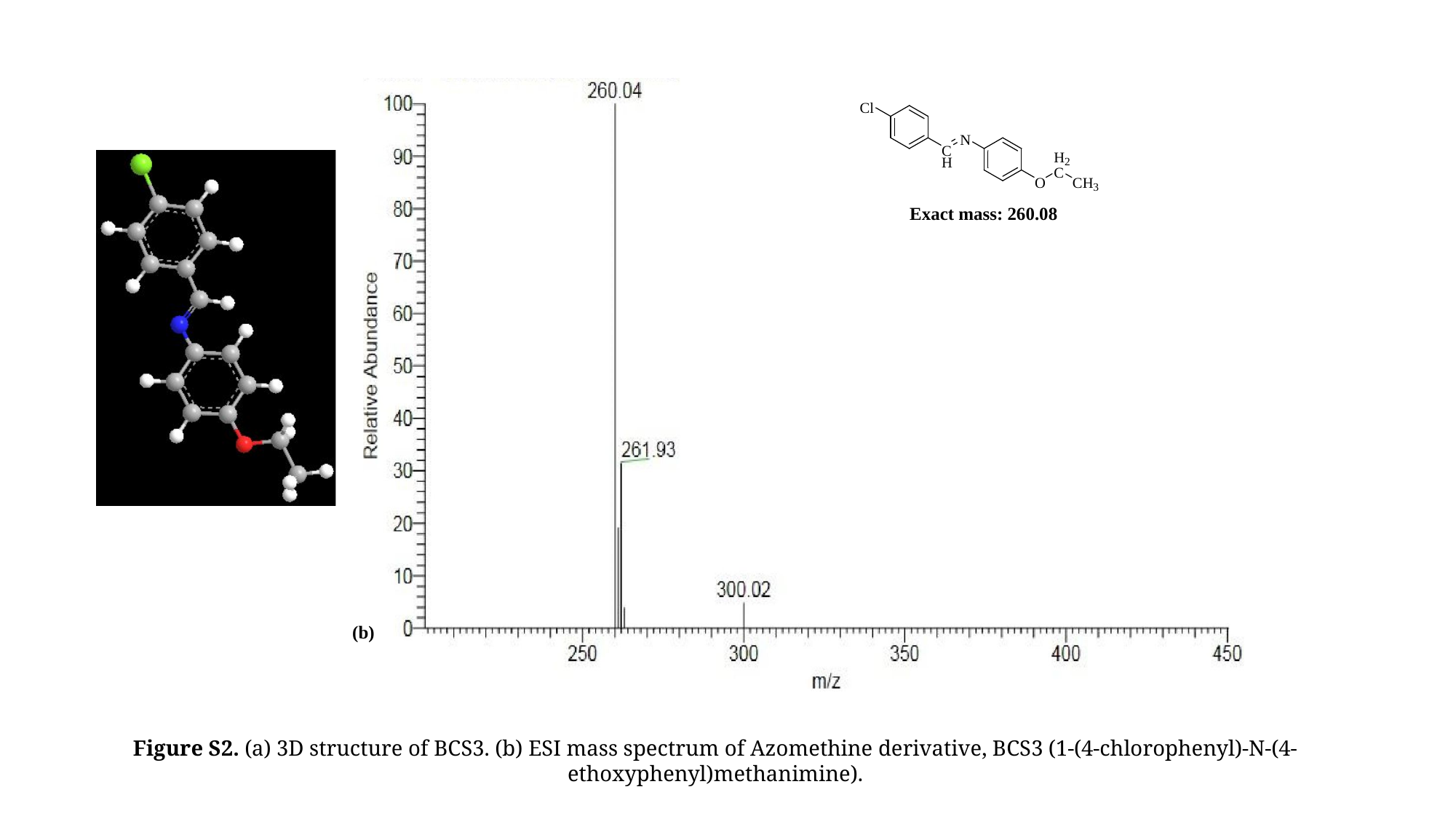

Exact mass: 260.08
(a)
(b)
Figure S2. (a) 3D structure of BCS3. (b) ESI mass spectrum of Azomethine derivative, BCS3 (1-(4-chlorophenyl)-N-(4-ethoxyphenyl)methanimine).

## Slide 5
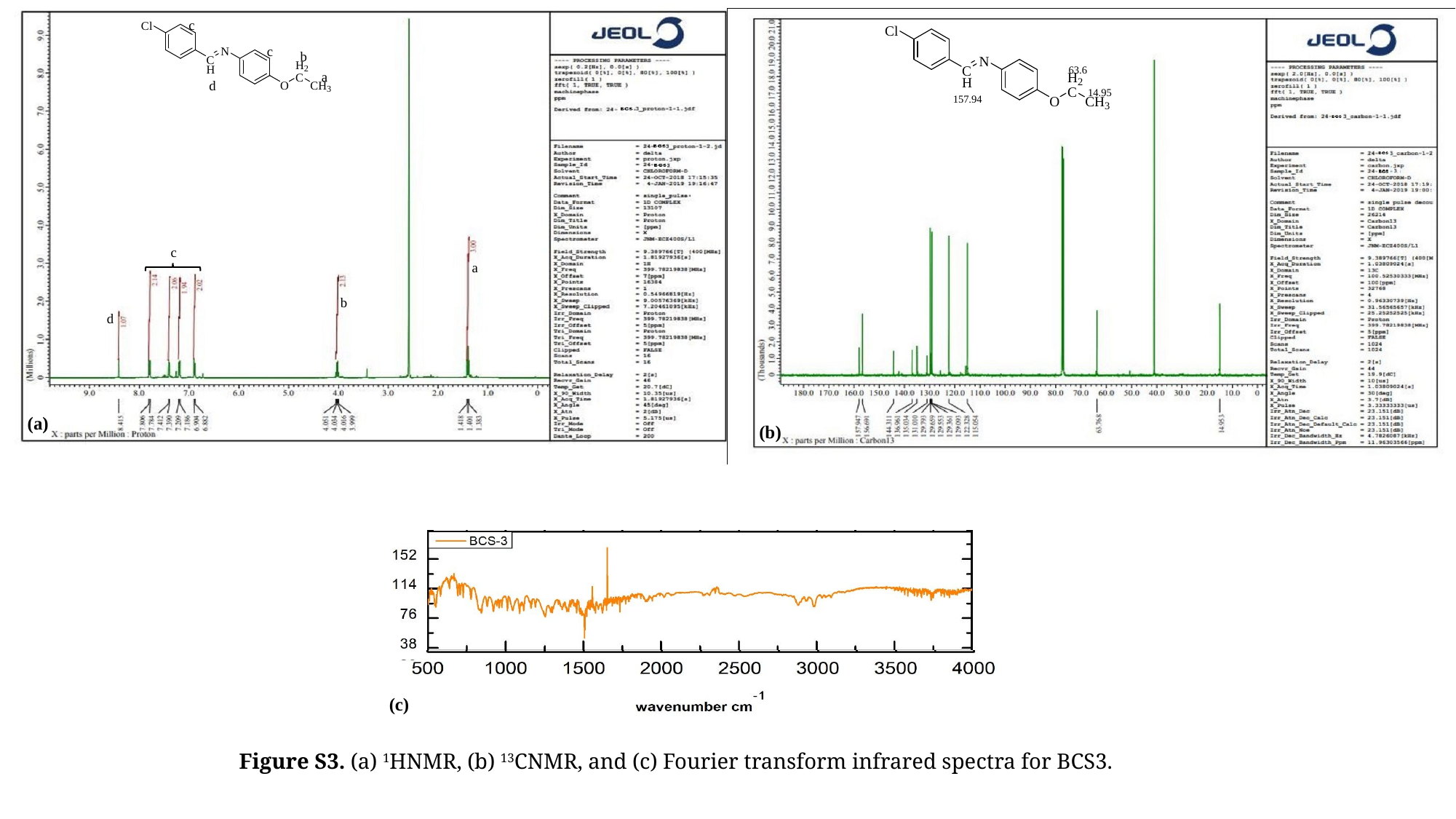

c
c
b
a
d
c
a
b
d
63.6
14.95
157.94
(a)
(B)
(b)
(c)
(c)
Figure S3. (a) 1HNMR, (b) 13CNMR, and (c) Fourier transform infrared spectra for BCS3.

## Slide 6
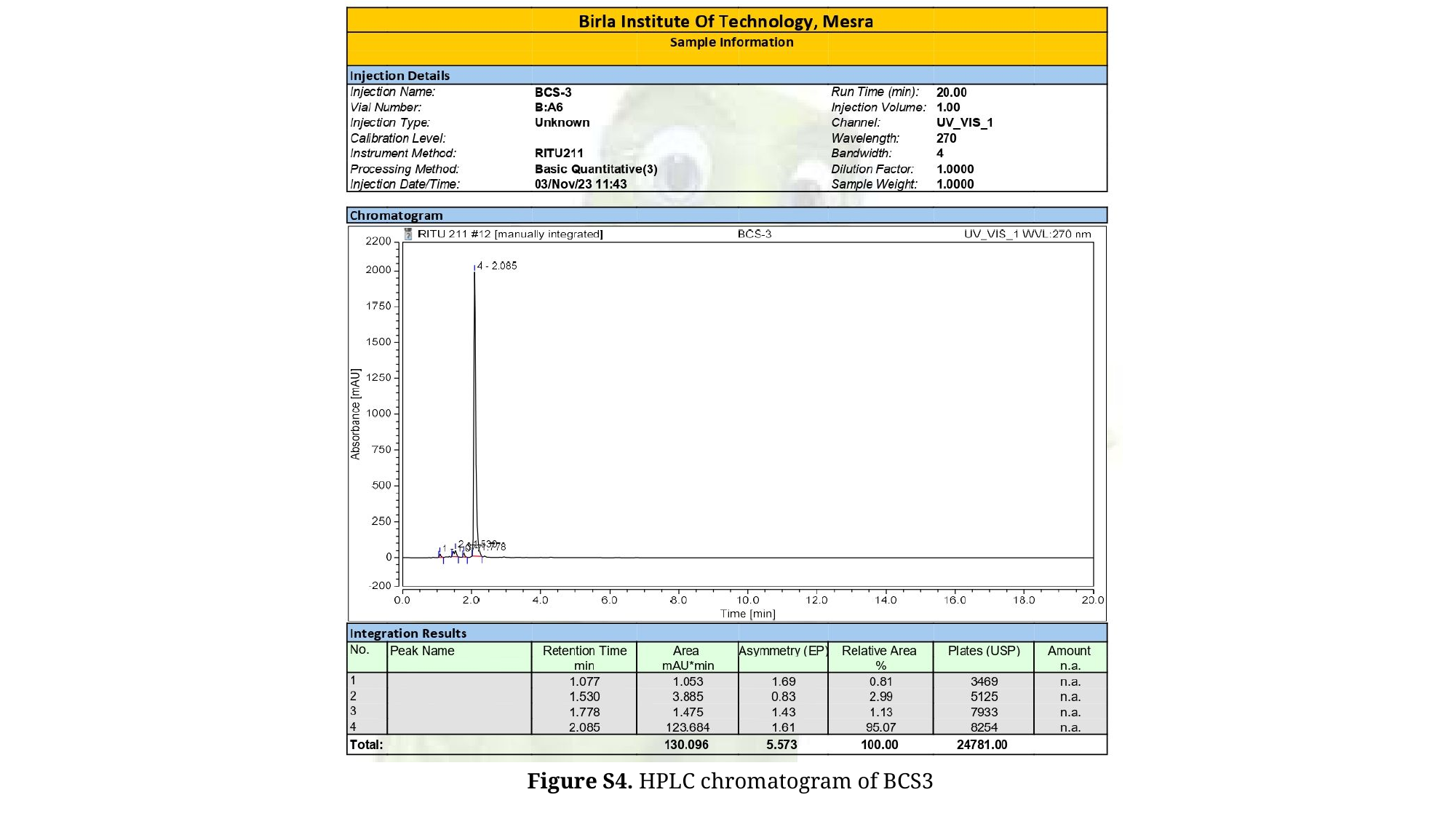

Figure S4. HPLC chromatogram of BCS3

## Slide 7
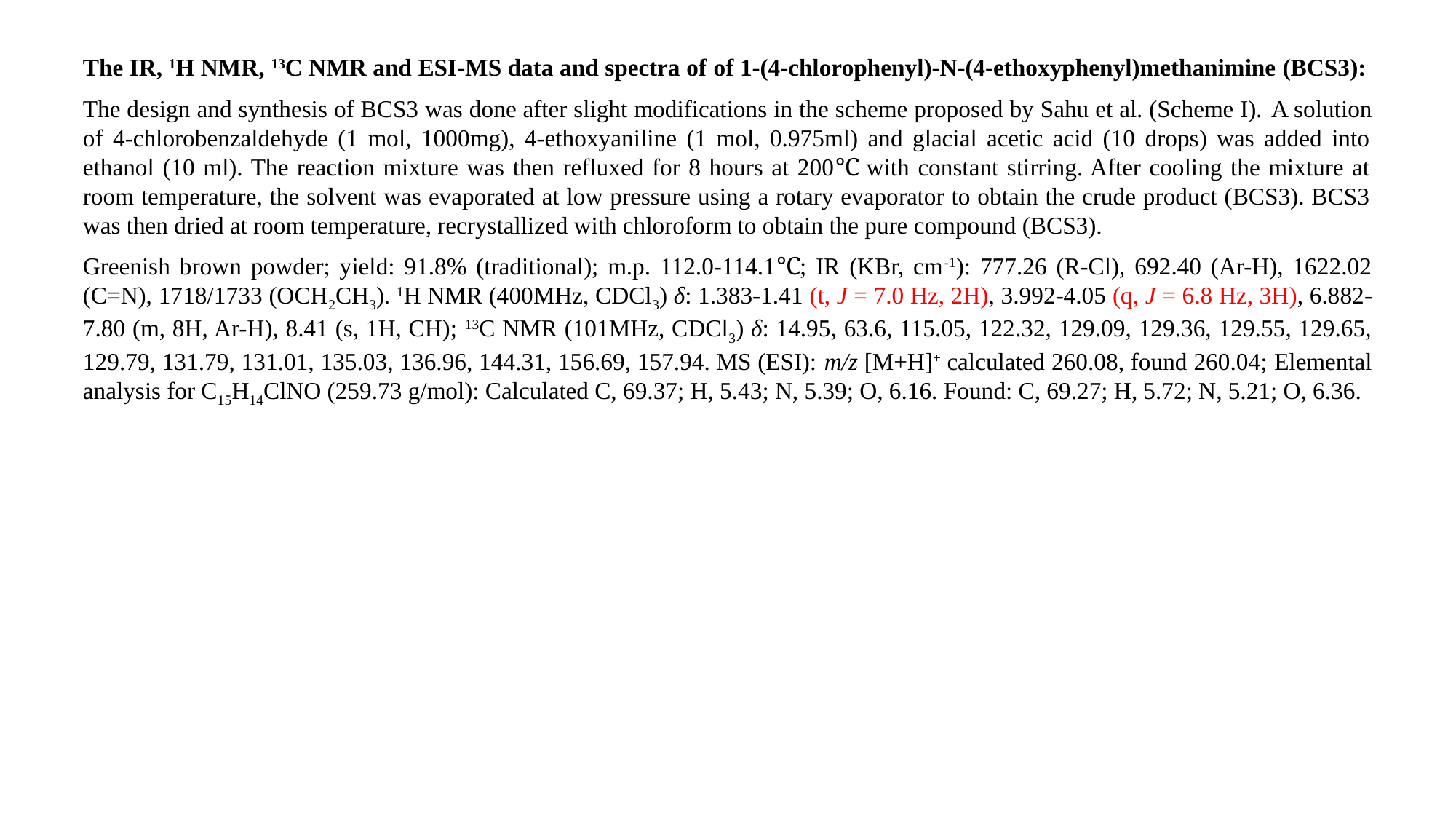

The IR, 1H NMR, 13C NMR and ESI-MS data and spectra of of 1-(4-chlorophenyl)-N-(4-ethoxyphenyl)methanimine (BCS3):
The design and synthesis of BCS3 was done after slight modifications in the scheme proposed by Sahu et al. (Scheme I). A solution of 4-chlorobenzaldehyde (1 mol, 1000mg), 4-ethoxyaniline (1 mol, 0.975ml) and glacial acetic acid (10 drops) was added into ethanol (10 ml). The reaction mixture was then refluxed for 8 hours at 200℃ with constant stirring. After cooling the mixture at room temperature, the solvent was evaporated at low pressure using a rotary evaporator to obtain the crude product (BCS3). BCS3 was then dried at room temperature, recrystallized with chloroform to obtain the pure compound (BCS3).
Greenish brown powder; yield: 91.8% (traditional); m.p. 112.0-114.1℃; IR (KBr, cm-1): 777.26 (R-Cl), 692.40 (Ar-H), 1622.02 (C=N), 1718/1733 (OCH2CH3). 1H NMR (400MHz, CDCl3) δ: 1.383-1.41 (t, J = 7.0 Hz, 2H), 3.992-4.05 (q, J = 6.8 Hz, 3H), 6.882-7.80 (m, 8H, Ar-H), 8.41 (s, 1H, CH); 13C NMR (101MHz, CDCl3) δ: 14.95, 63.6, 115.05, 122.32, 129.09, 129.36, 129.55, 129.65, 129.79, 131.79, 131.01, 135.03, 136.96, 144.31, 156.69, 157.94. MS (ESI): m/z [M+H]+ calculated 260.08, found 260.04; Elemental analysis for C15H14ClNO (259.73 g/mol): Calculated C, 69.37; H, 5.43; N, 5.39; O, 6.16. Found: C, 69.27; H, 5.72; N, 5.21; O, 6.36.

## Slide 8
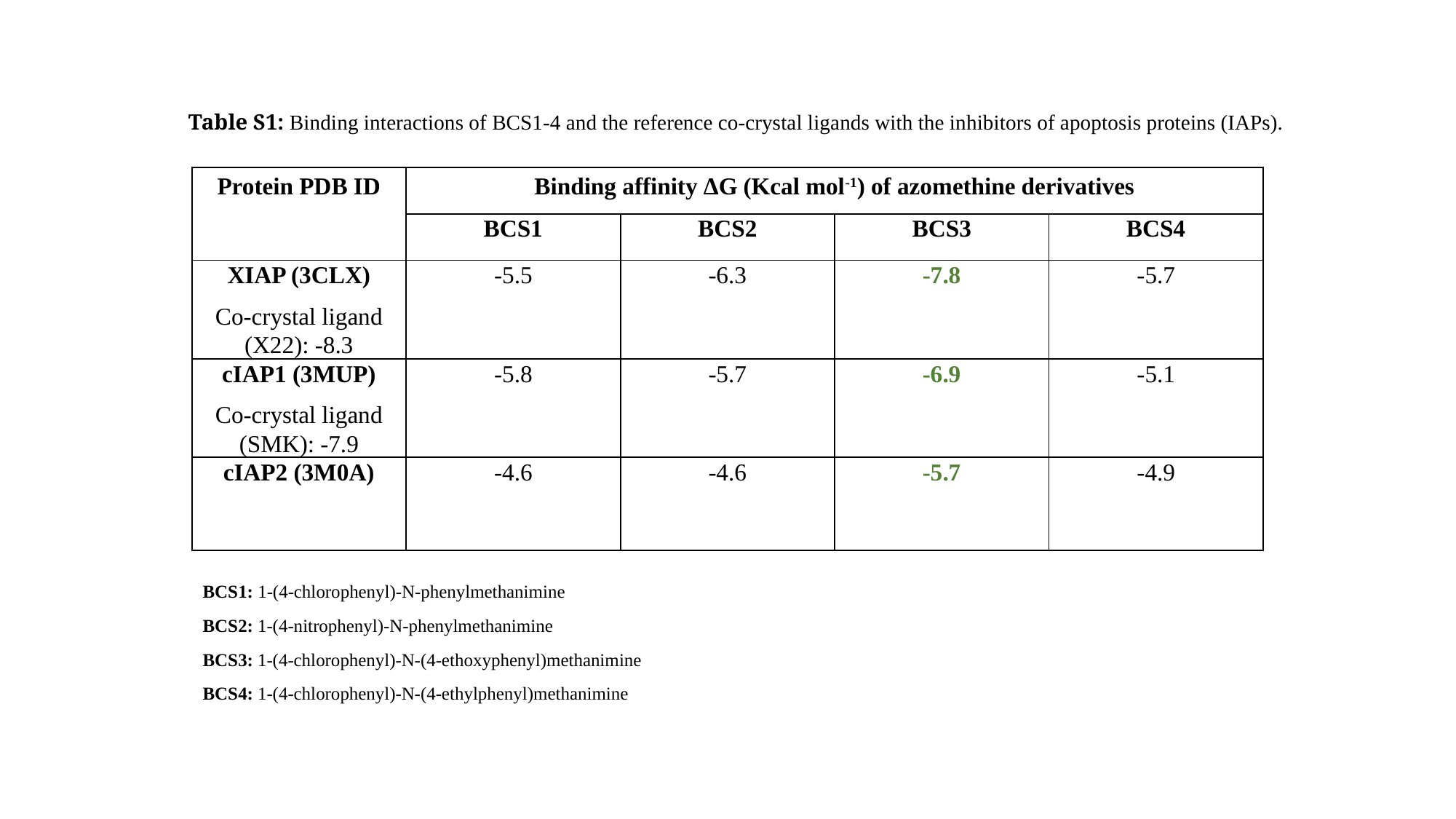

Table S1: Binding interactions of BCS1-4 and the reference co-crystal ligands with the inhibitors of apoptosis proteins (IAPs).
| Protein PDB ID | Binding affinity ΔG (Kcal mol-1) of azomethine derivatives | | | |
| --- | --- | --- | --- | --- |
| | BCS1 | BCS2 | BCS3 | BCS4 |
| XIAP (3CLX) Co-crystal ligand (X22): -8.3 | -5.5 | -6.3 | -7.8 | -5.7 |
| cIAP1 (3MUP) Co-crystal ligand (SMK): -7.9 | -5.8 | -5.7 | -6.9 | -5.1 |
| cIAP2 (3M0A) | -4.6 | -4.6 | -5.7 | -4.9 |
BCS1: 1-(4-chlorophenyl)-N-phenylmethanimine
BCS2: 1-(4-nitrophenyl)-N-phenylmethanimine
BCS3: 1-(4-chlorophenyl)-N-(4-ethoxyphenyl)methanimine
BCS4: 1-(4-chlorophenyl)-N-(4-ethylphenyl)methanimine

## Slide 9
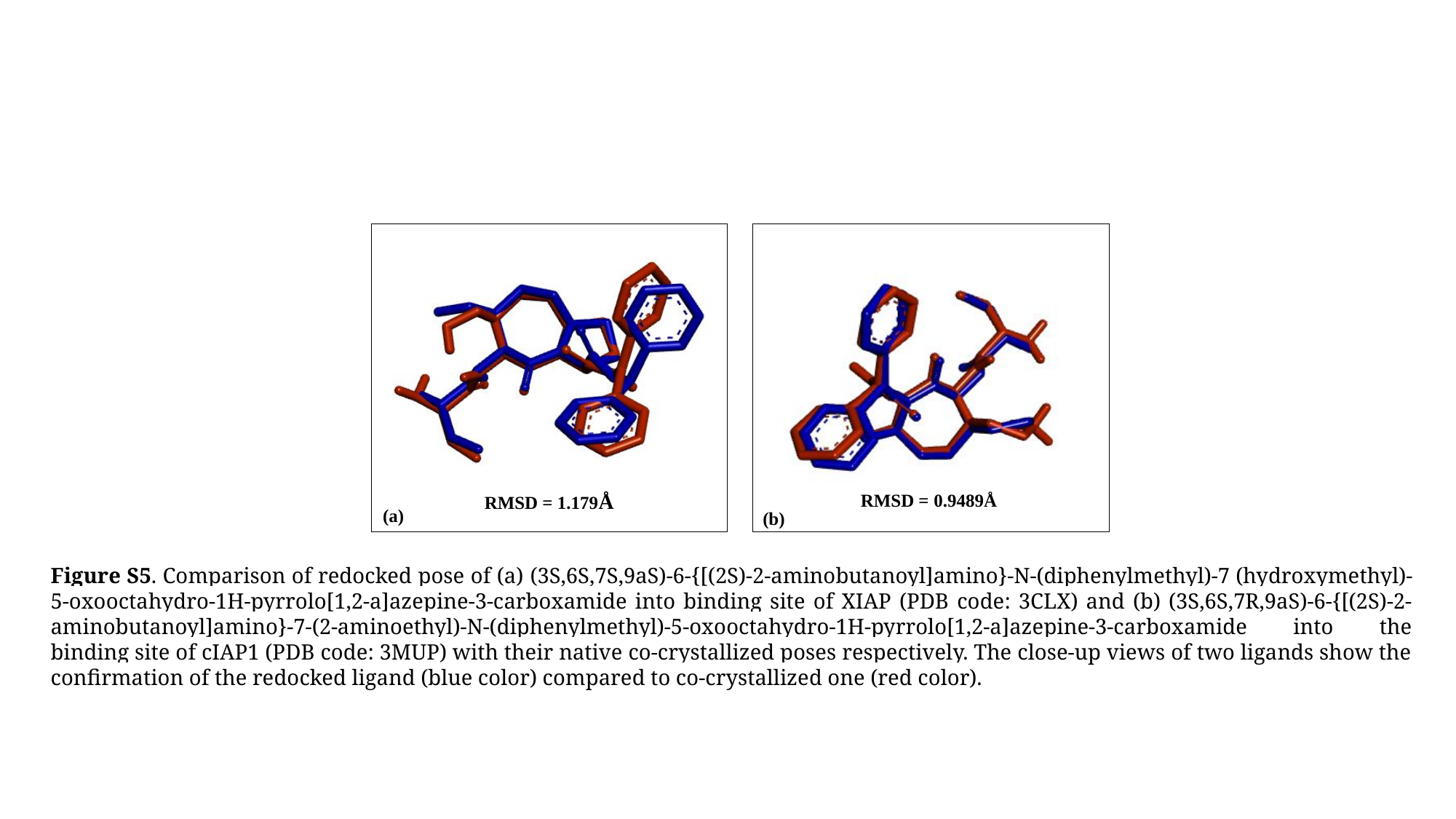

RMSD = 1.179Å
RMSD = 0.9489Å
(a)
(b)
Figure S5. Comparison of redocked pose of (a) (3S,6S,7S,9aS)-6-{[(2S)-2-aminobutanoyl]amino}-N-(diphenylmethyl)-7 (hydroxymethyl)-5-oxooctahydro-1H-pyrrolo[1,2-a]azepine-3-carboxamide into binding site of XIAP (PDB code: 3CLX) and (b) (3S,6S,7R,9aS)-6-{[(2S)-2-aminobutanoyl]amino}-7-(2-aminoethyl)-N-(diphenylmethyl)-5-oxooctahydro-1H-pyrrolo[1,2-a]azepine-3-carboxamide into the binding site of cIAP1 (PDB code: 3MUP) with their native co-crystallized poses respectively. The close-up views of two ligands show the confirmation of the redocked ligand (blue color) compared to co-crystallized one (red color).

## Slide 10
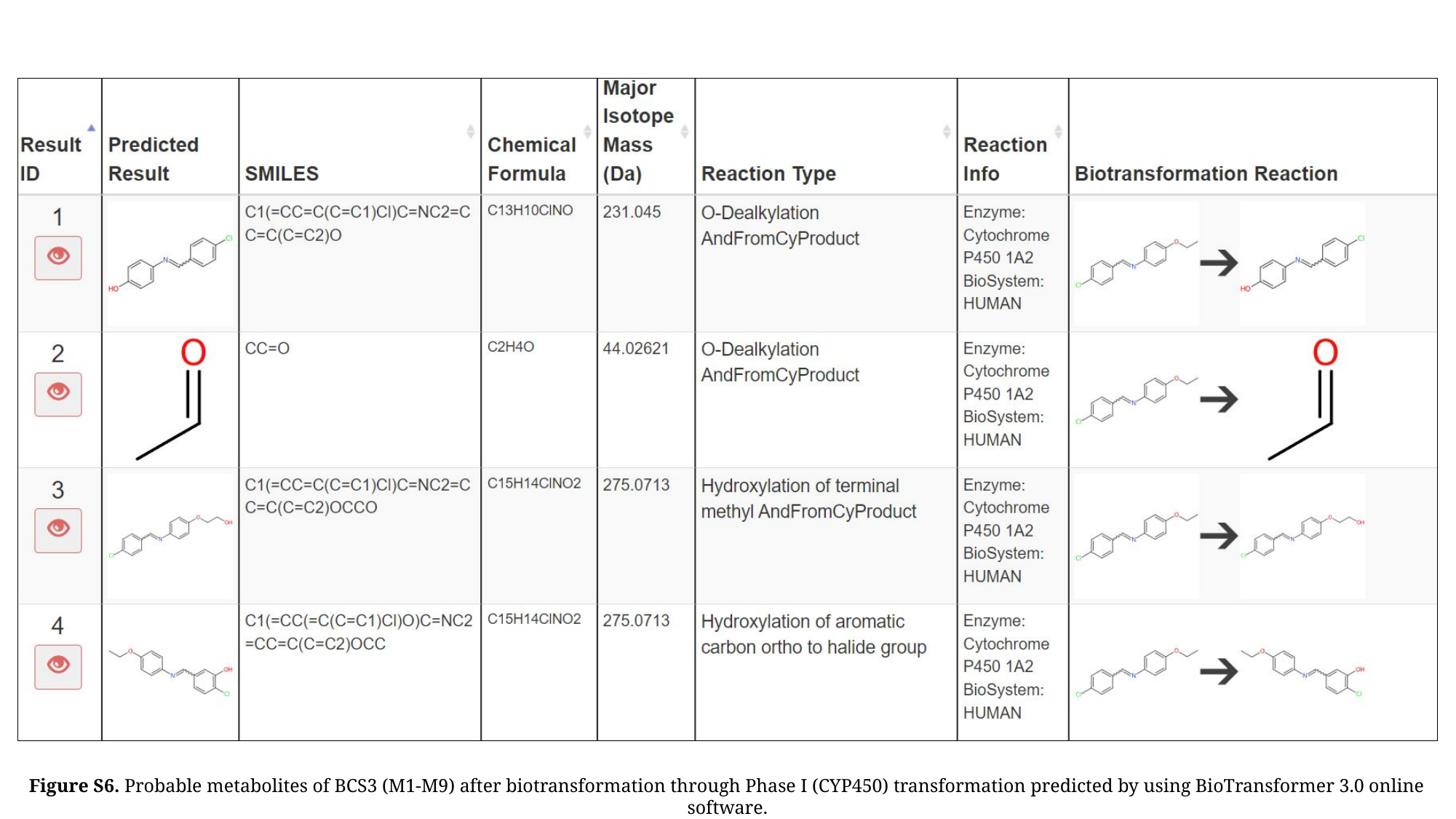

Figure S6. Probable metabolites of BCS3 (M1-M9) after biotransformation through Phase I (CYP450) transformation predicted by using BioTransformer 3.0 online software.

## Slide 11
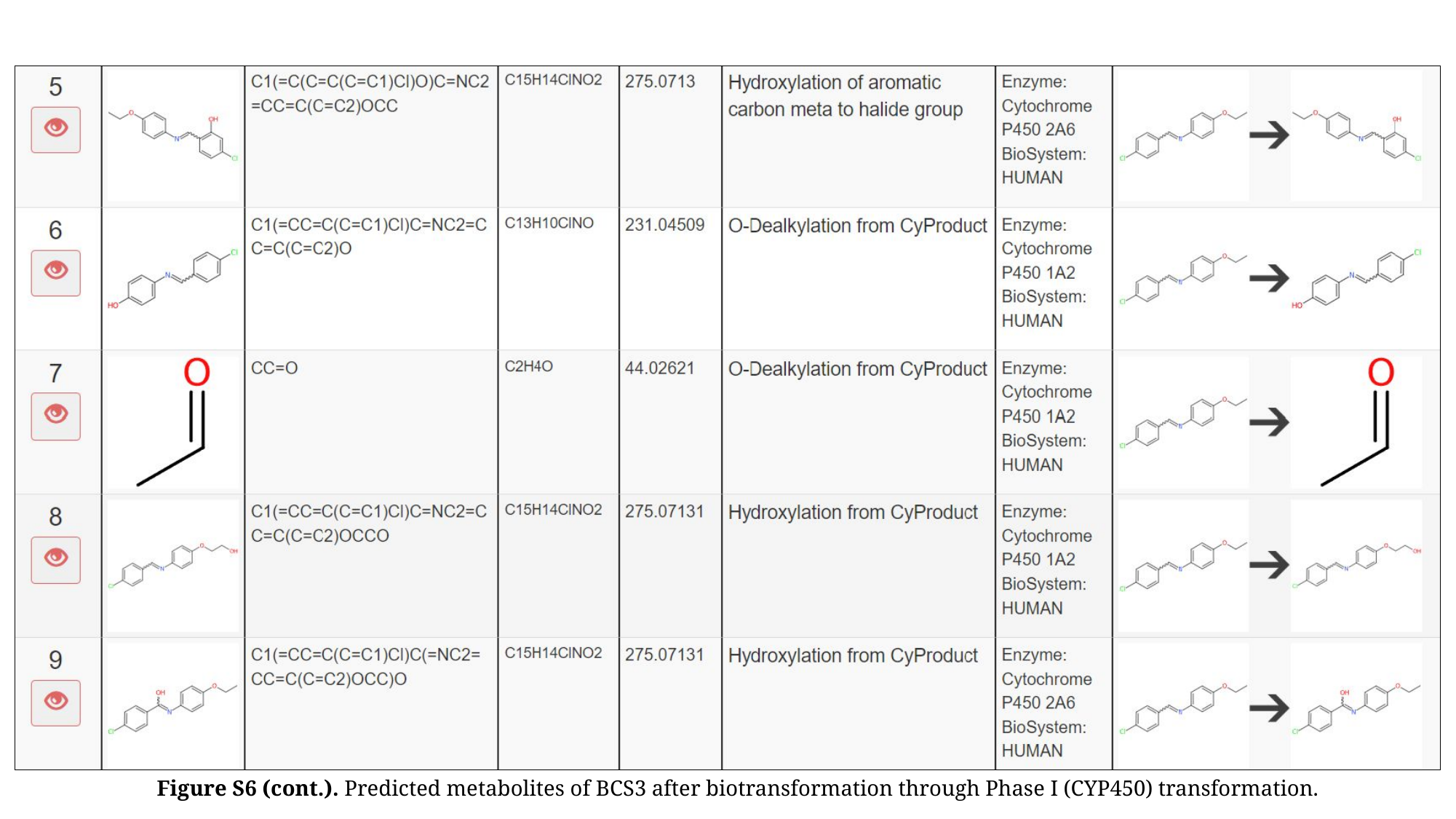

Figure S6 (cont.). Predicted metabolites of BCS3 after biotransformation through Phase I (CYP450) transformation.

## Slide 12
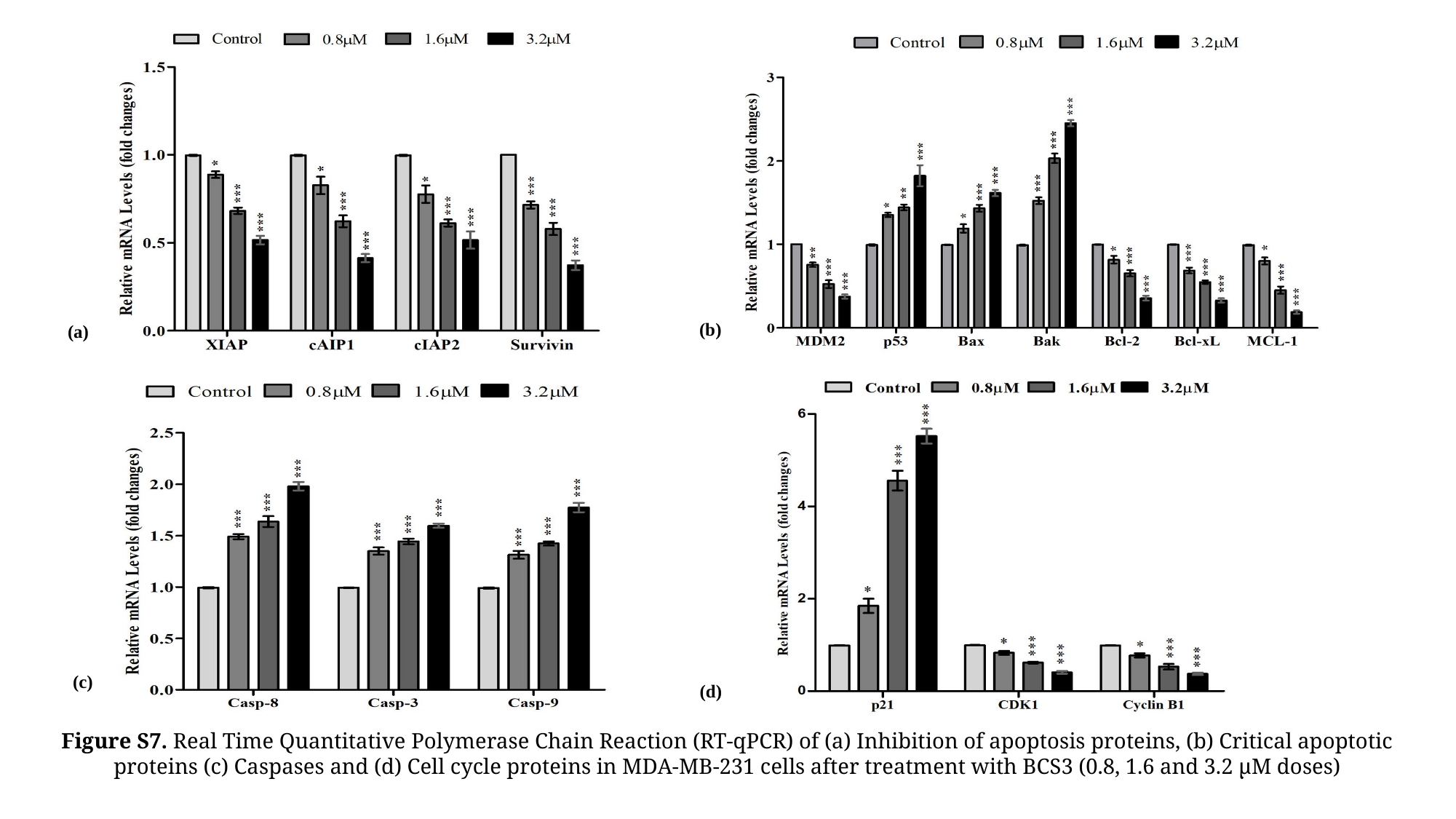

(b)
(a)
(c)
(d)
Figure S7. Real Time Quantitative Polymerase Chain Reaction (RT-qPCR) of (a) Inhibition of apoptosis proteins, (b) Critical apoptotic proteins (c) Caspases and (d) Cell cycle proteins in MDA-MB-231 cells after treatment with BCS3 (0.8, 1.6 and 3.2 µM doses)

## Slide 13
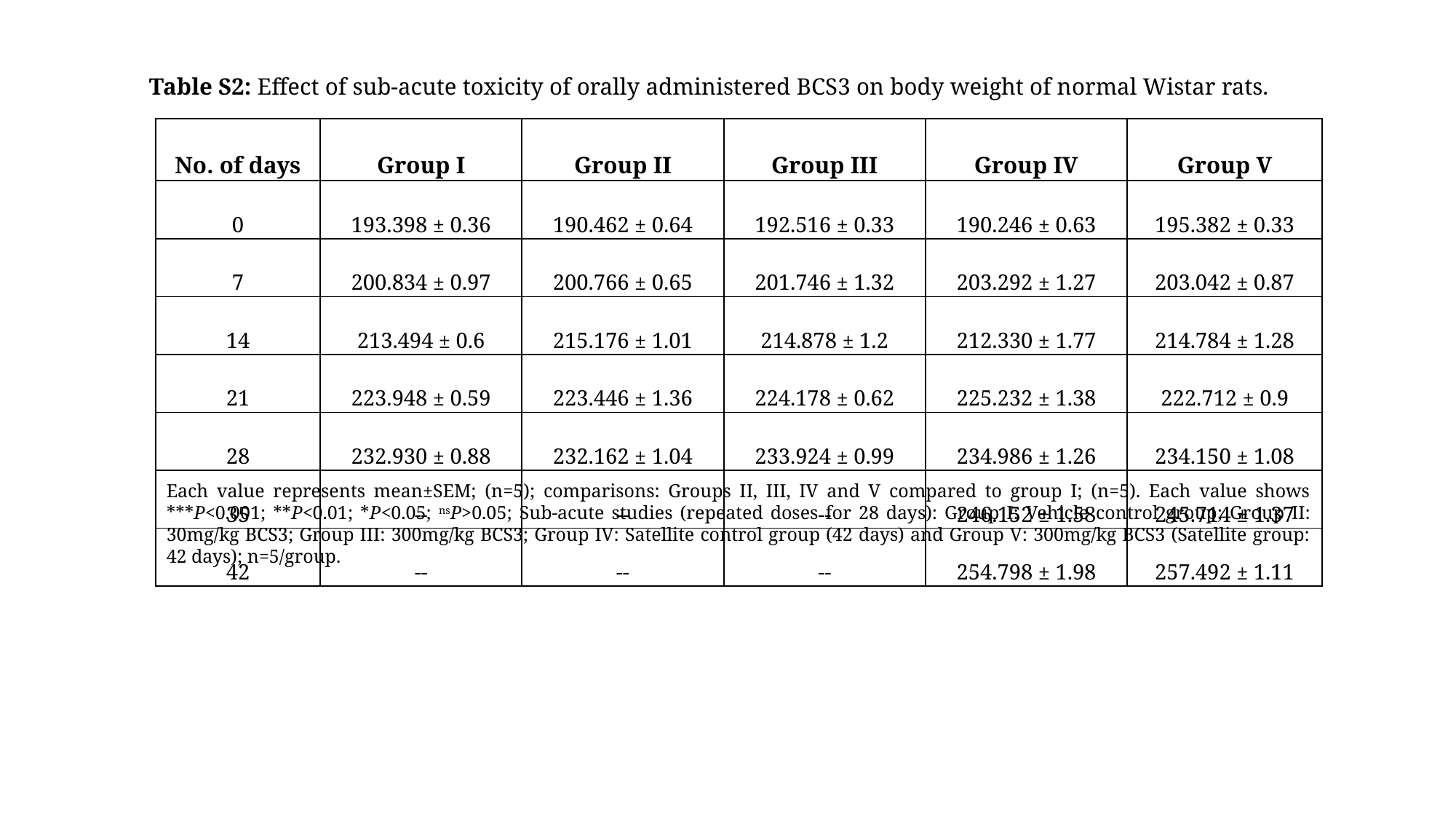

Table S2: Effect of sub-acute toxicity of orally administered BCS3 on body weight of normal Wistar rats.
| No. of days | Group I | Group II | Group III | Group IV | Group V |
| --- | --- | --- | --- | --- | --- |
| 0 | 193.398 ± 0.36 | 190.462 ± 0.64 | 192.516 ± 0.33 | 190.246 ± 0.63 | 195.382 ± 0.33 |
| 7 | 200.834 ± 0.97 | 200.766 ± 0.65 | 201.746 ± 1.32 | 203.292 ± 1.27 | 203.042 ± 0.87 |
| 14 | 213.494 ± 0.6 | 215.176 ± 1.01 | 214.878 ± 1.2 | 212.330 ± 1.77 | 214.784 ± 1.28 |
| 21 | 223.948 ± 0.59 | 223.446 ± 1.36 | 224.178 ± 0.62 | 225.232 ± 1.38 | 222.712 ± 0.9 |
| 28 | 232.930 ± 0.88 | 232.162 ± 1.04 | 233.924 ± 0.99 | 234.986 ± 1.26 | 234.150 ± 1.08 |
| 35 | -- | -- | -- | 246.152 ± 1.58 | 245.714 ± 1.37 |
| 42 | -- | -- | -- | 254.798 ± 1.98 | 257.492 ± 1.11 |
Each value represents mean±SEM; (n=5); comparisons: Groups II, III, IV and V compared to group I; (n=5). Each value shows ***P<0.001; **P<0.01; *P<0.05; nsP>0.05; Sub-acute studies (repeated doses for 28 days): Group I: Vehicle control group; Group II: 30mg/kg BCS3; Group III: 300mg/kg BCS3; Group IV: Satellite control group (42 days) and Group V: 300mg/kg BCS3 (Satellite group: 42 days); n=5/group.

## Slide 14
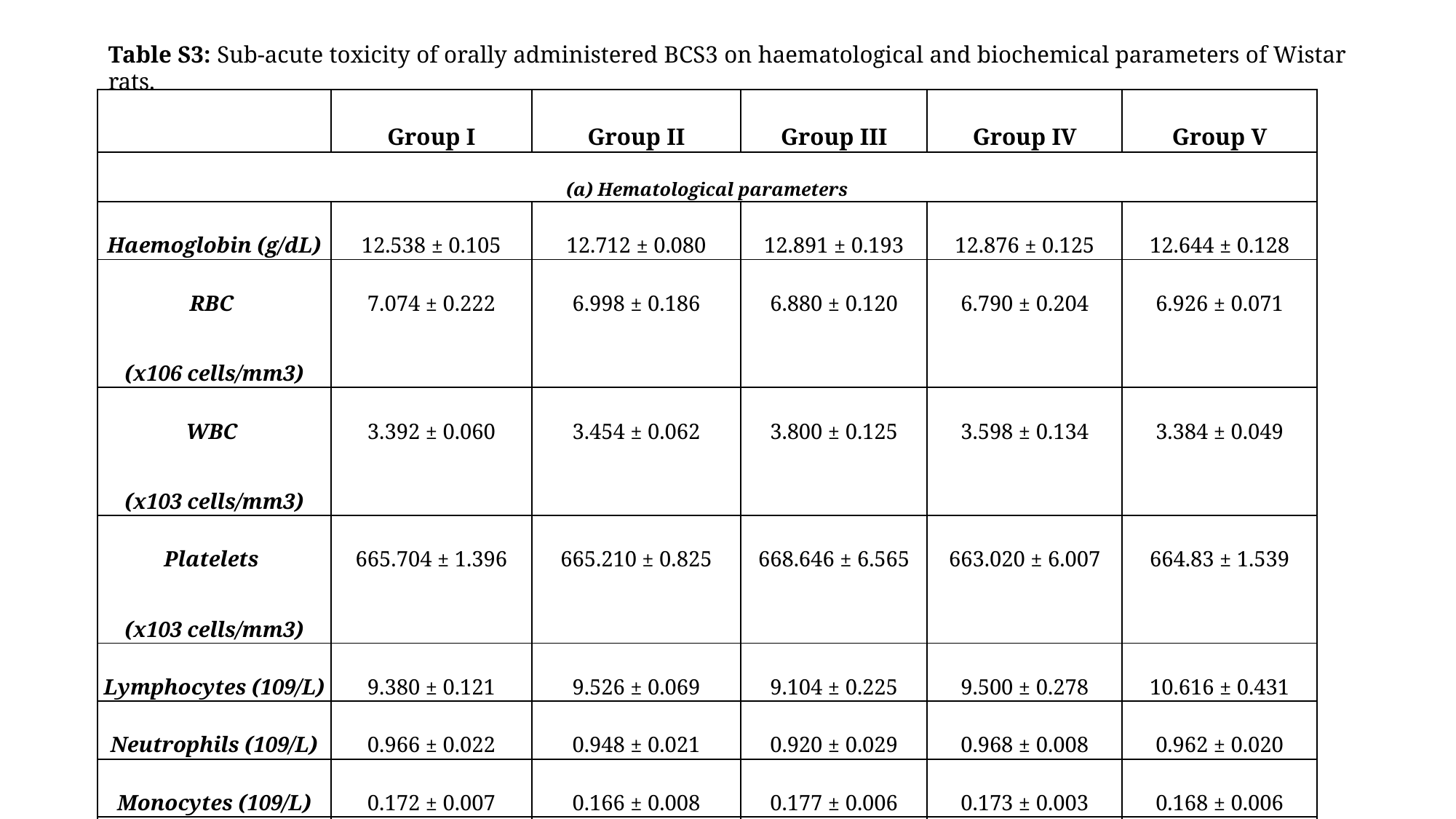

Table S3: Sub-acute toxicity of orally administered BCS3 on haematological and biochemical parameters of Wistar rats.
| | Group I | Group II | Group III | Group IV | Group V |
| --- | --- | --- | --- | --- | --- |
| (a) Hematological parameters | | | | | |
| Haemoglobin (g/dL) | 12.538 ± 0.105 | 12.712 ± 0.080 | 12.891 ± 0.193 | 12.876 ± 0.125 | 12.644 ± 0.128 |
| RBC (x106 cells/mm3) | 7.074 ± 0.222 | 6.998 ± 0.186 | 6.880 ± 0.120 | 6.790 ± 0.204 | 6.926 ± 0.071 |
| WBC (x103 cells/mm3) | 3.392 ± 0.060 | 3.454 ± 0.062 | 3.800 ± 0.125 | 3.598 ± 0.134 | 3.384 ± 0.049 |
| Platelets (x103 cells/mm3) | 665.704 ± 1.396 | 665.210 ± 0.825 | 668.646 ± 6.565 | 663.020 ± 6.007 | 664.83 ± 1.539 |
| Lymphocytes (109/L) | 9.380 ± 0.121 | 9.526 ± 0.069 | 9.104 ± 0.225 | 9.500 ± 0.278 | 10.616 ± 0.431 |
| Neutrophils (109/L) | 0.966 ± 0.022 | 0.948 ± 0.021 | 0.920 ± 0.029 | 0.968 ± 0.008 | 0.962 ± 0.020 |
| Monocytes (109/L) | 0.172 ± 0.007 | 0.166 ± 0.008 | 0.177 ± 0.006 | 0.173 ± 0.003 | 0.168 ± 0.006 |
| Eosinophils (109/L) | 0.085 ± 0.002 | 0.085 ± 0.001 | 0.086 ± 0.004 | 0.087 ± 0.003 | 0.085 ± 0.002 |
| Basophils (109/L) | 0.066 ± 0.002 | 0.067 ± 0.003 | 0.068 ± 0.003 | 0.069 ± 0.002 | 0.069 ± 0.002 |

## Slide 15
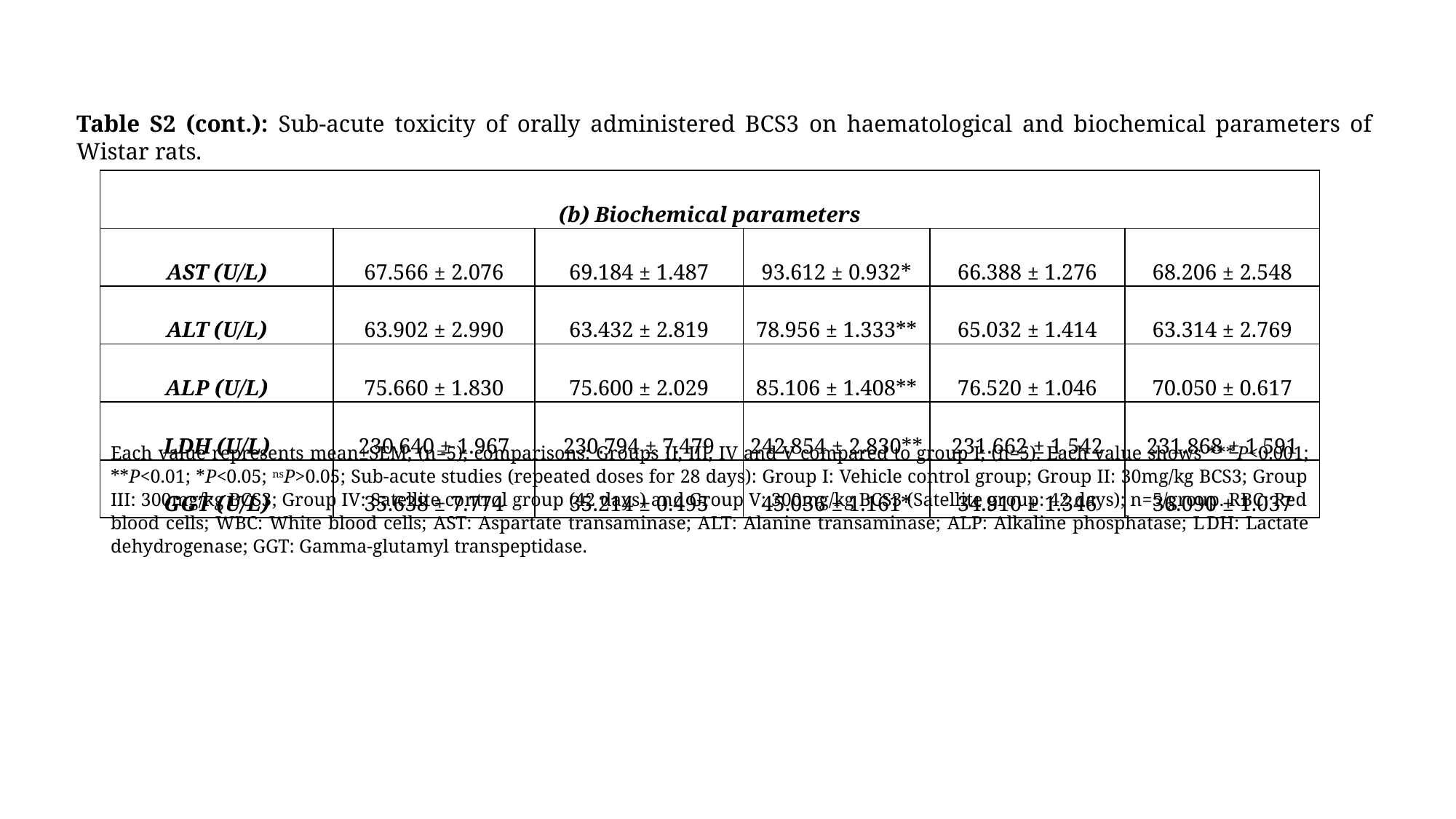

Table S2 (cont.): Sub-acute toxicity of orally administered BCS3 on haematological and biochemical parameters of Wistar rats.
| (b) Biochemical parameters | | | | | |
| --- | --- | --- | --- | --- | --- |
| AST (U/L) | 67.566 ± 2.076 | 69.184 ± 1.487 | 93.612 ± 0.932\* | 66.388 ± 1.276 | 68.206 ± 2.548 |
| ALT (U/L) | 63.902 ± 2.990 | 63.432 ± 2.819 | 78.956 ± 1.333\*\* | 65.032 ± 1.414 | 63.314 ± 2.769 |
| ALP (U/L) | 75.660 ± 1.830 | 75.600 ± 2.029 | 85.106 ± 1.408\*\* | 76.520 ± 1.046 | 70.050 ± 0.617 |
| LDH (U/L) | 230.640 ± 1.967 | 230.794 ± 7.479 | 242.854 ± 2.830\*\* | 231.662 ± 1.542 | 231.868 ± 1.591 |
| GGT (U/L) | 35.638 ± 7.774 | 35.214 ± 0.495 | 45.036 ± 1.161\* | 34.910 ± 1.346 | 36.090 ± 1.037 |
Each value represents mean±SEM; (n=5); comparisons: Groups II, III, IV and V compared to group I; (n=5). Each value shows ***P<0.001; **P<0.01; *P<0.05; nsP>0.05; Sub-acute studies (repeated doses for 28 days): Group I: Vehicle control group; Group II: 30mg/kg BCS3; Group III: 300mg/kg BCS3; Group IV: Satellite control group (42 days) and Group V: 300mg/kg BCS3 (Satellite group: 42 days); n=5/group. RBC: Red blood cells; WBC: White blood cells; AST: Aspartate transaminase; ALT: Alanine transaminase; ALP: Alkaline phosphatase; LDH: Lactate dehydrogenase; GGT: Gamma-glutamyl transpeptidase.
